# Single-cell RNA sequencing reveals the tumor microenvironment and facilitates strategic choices to circumvent treatment failure in a chemorefractory bladder cancer patient

**DOI:** 10.1186/s13073-020-00741-6

**Published:** 2020-05-27

**Authors:** Hye Won Lee, Woosung Chung, Hae-Ock Lee, Da Eun Jeong, Areum Jo, Joung Eun Lim, Jeong Hee Hong, Do-Hyun Nam, Byong Chang Jeong, Se Hoon Park, Kyeung-Min Joo, Woong-Yang Park

**Affiliations:** 1 Department of Anatomy and Cell Biology, Sungkyunkwan University School of Medicine, Suwon, 16419 Republic of Korea; 2 Single Cell Network Research Center, Sungkyunkwan University School of Medicine, Suwon, 16419 Republic of Korea; 3 Samsung Advanced Institute of Health Science and Technology, Sungkyunkwan University, Seoul, 06351 Republic of Korea; 4 Department of Molecular Cell Biology, Sungkyunkwan University School of Medicine, Suwon, 16419 Republic of Korea; 5 Department of Hospital Medicine, Yonsei University College of Medicine, Seoul, 03722 Republic of Korea; 6 Samsung Genome Institute, Samsung Medical Center, Seoul, 06351 Republic of Korea; 7 DCGen Co., Ltd, Seoul, 03170 Republic of Korea; 8 Department of Urology, Samsung Medical Center, Sungkyunkwan University School of Medicine, Seoul, 06351 Republic of Korea; 9 Department of Urology, Dankook University College of Medicine, Cheonan, 31116 Republic of Korea; 10 Department of Neurosurgery, Samsung Medical Center, Sungkyunkwan University School of Medicine, Seoul, 06351 Republic of Korea; 11 Division of Hematology and Oncology, Department of Medicine, Samsung Medical Center, Sungkyunkwan University School of Medicine, Seoul, 06351 Republic of Korea; 12 Stem Cell and Regenerative Medicine Center, Research Institute for Future Medicine, Samsung Medical Center, Seoul, 06351 Republic of Korea

**Keywords:** Treatment resistance, Tumoral heterogeneity, Tumor microenvironment, Single-cell RNA sequencing, Muscle-invasive urothelial bladder cancer

## Abstract

**Background:**

Tumor cell-intrinsic mechanisms and complex interactions with the tumor microenvironment contribute to therapeutic failure via tumor evolution. It may be possible to overcome treatment resistance by developing a personalized approach against relapsing cancers based on a comprehensive analysis of cell type-specific transcriptomic changes over the clinical course of the disease using single-cell RNA sequencing (scRNA-seq).

**Methods:**

Here, we used scRNA-seq to depict the tumor landscape of a single case of chemo-resistant metastatic, muscle-invasive urothelial bladder cancer (MIUBC) addicted to an activating Harvey rat sarcoma viral oncogene homolog (*HRAS*) mutation. In order to analyze tumor evolution and microenvironmental changes upon treatment, we also applied scRNA-seq to the corresponding patient-derived xenograft (PDX) before and after treatment with tipifarnib, a HRAS-targeting agent under clinical evaluation.

**Results:**

In the parallel analysis of the human MIUBC and the PDX, diverse stromal and immune cell populations recapitulated the cellular composition in the human and mouse tumor microenvironment. Treatment with tipifarnib showed dramatic anticancer effects but was unable to achieve a complete response. Importantly, the comparative scRNA-seq analysis between pre- and post-tipifarnib-treated PDX revealed the nature of tipifarnib-refractory tumor cells and the tumor-supporting microenvironment. Based on the upregulation of programmed death-ligand 1 (PD-L1) in surviving tumor cells, and the accumulation of multiple immune-suppressive subsets from post-tipifarnib-treated PDX, a PD-L1 inhibitor, atezolizumab, was clinically applied; this resulted in a favorable response from the patient with acquired resistance to tipifarnib.

**Conclusion:**

We presented a single case report demonstrating the power of scRNA-seq for visualizing the tumor microenvironment and identifying molecular and cellular therapeutic targets in a treatment-refractory cancer patient.

## Background

The precise identification of truncal driver mutations is essential for minimizing drug resistance or tumor relapse with targeted therapeutics for refractory, rapidly progressive cancers [1–3]. However, specific targeted therapies constitute an often stringent, directional selection pressure on distinct subclones with either intrinsic or acquired resistance due to the presence of multiple genotypically and/or phenotypically distinct populations affecting diverse signal transduction pathways within a single tumor [2, 4]. Additionally, various types of tumor-associated stromal cells and the extracellular milieu within the tumor microenvironment (TME) play key roles in governing the plasticity of the phenotypic traits of cancer cells, as well as in mediating the response to selection pressure [2, 5–8]. Significantly more work is required in order to achieve an understanding of an altered and extremely plastic interactive feedback loop between cancer cells and the TME for the design of effective therapeutic interventions, as well as how this might alter the current combination treatment strategies.

Unfortunately, conventional bulk next-generation sequencing techniques have limitations in their ability to resolve tumor subpopulations and the TME [9]; this is in addition to the technical difficulties in preparing cancer cells and TME cells obtained during serial and multisite sampling over the clinical course of treatment. Recently developed single-cell RNA sequencing (scRNA-seq) technologies allow high-resolution characterization of distinct gene modules using a relatively small number of cells [6, 10–16]. This technology allows dissection of the critical drug target pathways activated in heterogeneous tumor cells that remain after treatment and from diverse tumor-associated non-malignant cells within the surrounding activated stroma in order to design tailored combination therapy targeting both tumor cells and the associated TME [6, 10–16].

Muscle-invasive urothelial bladder cancers (MIUBCs) are clinically aggressive and fatal, with a 5-year relative survival rate of 5%, owing to a high probability of systemic dissemination and a lack of improved therapeutic guidelines [17, 18]. In particular, intratumoral and intertumoral heterogeneity (genetic, molecular, and microenvironmental) of MIUBCs [19–23] necessitates the design of personalized interventions against tumor cell-intrinsic mechanisms and complex interactions, with the TME contributing to the therapeutic failure and tumor evolution of MIUBCs. Therefore, there is a need to monitor the evolutionary trajectories of tumor cells and the surrounding TME using pre- and post-treatment samples in order [2, 4–8]. Previously, we successfully resolved critical molecular pathways in individual tumor cells and associated these pathways with therapeutic outcomes using patient-derived tumor xenografts (PDXs) [10, 11]. Stromal and innate immune cell components in PDXs may recapitulate the TME in human tumor tissues [24, 25], and species-specific analysis based on PDX models is one of the easiest ways to distinguish the TME (mouse transcriptome) from tumor cells (human transcriptome) [26]. In this study, we applied scRNA-seq to resected primary tumor and PDX from a single patient with chemotherapy-resistant metastatic MIUBC for the in-depth analysis of multiple mechanisms underlying treatment-refractory cancers. Comparative scRNA-seq of tumor cells and the TME between the primary tumor and corresponding PDX provided important clues to develop a sequential option to circumvent tumor progression after targeting oncogene addiction, with successful translation in the patient.

## Methods

### Patient and tumor samples

This study was approved by the Institutional Review Board (IRB) of the Samsung Medical Center, Seoul, Korea (IRB No. 201004004), and a single patient provided signed informed consent for the collection of specimens and detailed analyses of the derived genetic material, as well as for the participation in a phase 2 tipifarnib trial (ClinicalTrials.gov, NCT02535650). Each biopsied parental tumor mass was either chopped into fragments and frozen (BC159-T#3) or placed in formalin and embedded in paraffin for later analyses (BC159-T#1, BC159-T#2, and BC159-T#3). A blood pellet was used for the extraction of germline DNA. Fresh BC159-T#3 tumor tissue was stored on ice in Hank’s balanced salt solution (Gibco, Grand Island, NY, USA) supplemented with penicillin/streptomycin (Gibco) for transportation.

### In vivo validation of the targeted drug using established BC159-T#3 PDX

All mouse procedures were carried out according to the National Institute of Health Guidelines for the Care and Use of Laboratory Animals, and the protocol was approved by the IRB at the Samsung Medical Center (No. 20170720002). For the establishment of BC159-T#3 PDX, BC159-T#3 tumor tissue was minced into approximately 1-mm^3^ fragments in a high-concentration Matrigel™ basement membrane matrix (BD Biosciences, Franklin Lakes, NJ, USA) and directly implanted into the subcutaneous space of female 6- to 8-week-old BALB/c nude mice purchased from Orient Bio (Seoul, Korea). PDX tumors were harvested and divided into three samples for the generation of second-passage in vivo xenograft tumors, DNA/RNA extraction, and histopathological examination. The origin of each xenograft was validated by short tandem repeat DNA fingerprinting.

For an in vivo efficacy test of tipifarnib, 1 × 10^5^ dissociated BC159-T#3 PDX tumor cells were mixed 1:1 with Matrigel and inoculated subcutaneously into the right flank of each mouse. The tumor diameter was measured with calipers twice a week, and the tumor volume was calculated using the following formula: tumor volume (mm^3^) = (*l* × *w*^2^)/2, where *l* is the longest diameter of the tumor and *w* is the shortest diameter of the tumor. Mice bearing established tumors (100–150 mm^3^) were randomly allocated to a tipifarnib (50 mg/kg, oral gavage, twice a day) group and a vehicle control group and treated for 20 days. Throughout the study, the mice were weighed, and the tumor burden was monitored every 3 days. The mean tumor volumes were calculated for each group, and tumor growth curves were generated as a function of time. Tumors from each group were collected at the end of the experiment for further analysis.

### Immunohistochemistry (IHC) and measurement of proliferation and apoptosis in PDX

Tumors from the patient and PDX were embedded in paraffin, sectioned at 4 μm, and stained with hematoxylin and eosin. For immunochemical staining, formalin-fixed, paraffin-embedded sections were deparaffinized and rehydrated [10, 11]. Heat-induced epitope retrieval was performed using a target retrieval solution (Dako, Glostrup, Denmark) for 20 min in a microwave oven. Slides were treated with 3% hydrogen peroxide for 12 min to inactivate endogenous peroxidase and then blocked for 1 h at room temperature (RT) in a blocking solution (Dako). After blocking, the slides were incubated with primary antibodies, including mouse monoclonal antibodies against the HRASQ61R mutant (reactive to NRAS and HRAS, Spring Bioscience, Pleasanton, CA, USA), cytokeratin (CK) 5/6 (Dako), CK13 (Abcam, Paris, France), CK14 (Abcam), phosphorylated (p)-extracellular signal-regulated kinase (ERK) (Cell Signaling Technology, MA, USA), p-protein kinase B (AKT) (Abcam), α-smooth muscle actin (Dako), CD4 (Abcam), CD8 (Abcam), CD68 (Abcam), and programmed death-ligand 1 (PD-L1) (Abcam). After washing, the slides were incubated with secondary antibodies for 1 h at RT and counterstained with hematoxylin (Vector). Markers for proliferation and apoptosis were assessed by IHC. Proliferation was assessed using Ki-67 (BD Pharmingen), and apoptosis was determined by terminal deoxynucleotidyl transferase-mediated dUTP nick-end labeling (TUNEL) staining of the tumor sections using the DeadEnd™ colorimetric TUNEL system (Promega, Madison, WI, USA) [10, 11]. The proliferative and apoptotic indexes were calculated as a ratio of Ki-67-positive or TUNEL-positive cells to the total cell number, respectively, in high-power (× 400) fields.

### Whole exome sequencing (WES) and data processing

WES and data processing were performed as previously described [16]. Briefly, genomic DNA was extracted from the bulk tumor and whole blood using the QIAamp® DNA mini kit (Qiagen, Germantown, MD, USA) and QIAamp DNA blood maxi kit (Qiagen), respectively. Exome sequences were enriched using the SureSelect XT Human All Exon V5 kit (Agilent, Santa Clara, CA, USA) and sequenced in the 100-bp paired-end mode on the HiSeq 2500 system (Illumina, San Diego, CA, USA). The tumor and matched blood DNA were sequenced to 100× and 50× coverages, respectively. The sequencing reads were mapped to the human genome build hg19/GRCh37 with BWA-0.7.10 [27]. Aligned reads were realigned for known insertions or deletions, and their base-quality scores were recalibrated using GATK-3.2 modules with known variant sites identified from phase I of the 1000 Genomes Project (http://www.1000genomes.org/) and dbSNP-137 (http://www.ncbi.nlm.nih.gov/SNP/). MuTect-1.1.5 was used with default parameters to detect somatic SNVs, and mutations were annotated using Oncotator [28]. Additionally, the Control-FREEC package [29] was used to detect copy-number variations (CNVs), and CNVs with a *P* < 0.05 (Wilcoxon rank-sum test) were obtained. Druggable targeting genetic alterations were annotated from the OncoKB database (http://oncokb.org).

### Inferred CNVs

Low-expression genes (average expression level < 0.2) were discarded from the final gene expression matrix to reduce the noise, and the refined data were mean centered based on the average expression values of each gene. After all genes were sorted by their chromosomal position following *Z*-score normalization of each gene, inferred CNVs were calculated from the moving averages of 100 genes as the slide window size and adjusted as centered values across genes, as previously described [16]. Because of the possibility of distorting the moving average of particular genes, we restricted the centered gene expression to an absolute value │3│. To distinguish tumor cells from non-tumor cells, CNV scores for each cell were defined as the mean of squares of inferred CNV values across genes, and CNV correlations were defined as the correlation between each cell with average inferred CNV values of top 5% CNV scores. With two cutoffs of 0.02 of the CNV score and 0.2 of the CNV correlation, we assigned cells to tumor cells if the two measured values were above the two cutoffs and to non-tumor cells if the two measured values were below the two cutoffs. The remaining cells were assigned as undetermined cells. Finally, recalibration of inferred CNVs was performed in order to apply non-tumor cells as references [30].

### Whole transcriptome sequencing (WTS) and data processing

WTS and data processing were performed as previously described [16]. Briefly, WTS libraries were generated using the TruSeq RNA sample preparation v2 kit (Illumina) and sequenced on the HiSeq 2500 system (Illumina) on the 100-bp paired-end mode. The RNA reads were aligned to the human reference genome (hg19) using the STAR aligner with default parameters, and relative gene expression levels were quantified as transcripts per million (TPM) values using RSEM v1.2.17 with default parameters [31]. TPM values were summed up to adjust the isoform expression levels for each gene. For downstream analysis, TPM values < 1 were substituted with zero and log_2_-transformed after adding a value of one.

MIUBC can be segregated into at least five molecular subtypes distinguishable by combinatorial molecular signatures and divergent clinical outcomes [32–34]. In the present study, the molecular subtypes were assigned using the five subtypes from The Cancer Genome Atlas (TCGA) 2017 mRNA dataset [32]. After genes of the total matrix were restricted to the subtype signature gene list, we computed the average expression of signature genes for each subtype. Thereafter, a correlation was calculated between the bulk samples and each subtype. Finally, the highest value of Pearson’s correlation coefficient with a significant *P* value was defined as the molecular subtype of the bulk sample.

### Acquisition of TCGA-urothelial bladder carcinoma (TCGA-BLCA) data

A processed public WTS dataset with clinical information for TCGA-BLCA [32] was downloaded from the Firehose website (http://gdac.broadinstitute.org/). A total of 408 tumor samples with adequate clinical information were used for downstream analysis after merging with our bulk data in TPM values.

### Single-cell western analysis

In total, 1 mL of cell suspension (1 × 10^5^ cells) was loaded onto a standard scWest chip for 5 min. Optical visualization was performed in order to confirm and mark single-cell capture sites, followed by gentle washing in 1× suspension buffer (ProteinSimple, San Jose, CA, USA). The scWest chip was then submitted to the Milo system (ProteinSimple) for lysis (10 s), electrophoretic separation (60 s, 240 V), and protein immobilization (240 s). Protein targets were probed on-chip for 2 h at RT with primary antibodies, including goat anti-neuroblastoma RAS viral (v-ras) oncogene homolog (NRAS) (Abcam), mouse anti-p-ERK (Cell Signaling Technology, Beverly, MA, USA), rabbit anti-p-AKT (R&D Systems, Minneapolis, MN, USA), rabbit anti-histone H3 (Cell Signaling Technology), rabbit anti-NRASQ61R (Spring Bioscience), and mouse anti-vimentin (Dako), and then for 1 h at RT with secondary antibodies (Thermo Fisher Scientific, Waltham, MA, USA), including donkey anti-goat IgG, Alexa Fluor 488; donkey anti-mouse IgG, Alexa Fluor 555; and donkey anti-rabbit IgG, Alexa Fluor 647. The probed chip was washed, air-dried, and analyzed using GenePix 4400A Scanners (Molecular Devices, San Jose, CA, USA).

### Droplet-based scRNA-seq

In order to perform scRNA-seq, the primary sample (BC159-T#3) was directly obtained from the operating room, and PDX was generated from this primary sample. Specimens were dissociated into single cells according to previously published protocols [11, 35]. After resuspension in 1× phosphate-buffered saline, all single-cell suspensions were loaded into a 10x Chromium Controller (10x Genomics, Pleasanton, CA, USA), aiming for 7000 cells, with the Single Cell 3′ v2 reagent kit (10x Genomics), according to the manufacturer’s instructions. Following Gem capturing and lysis, cDNA was synthesized and amplified to construct sequencing libraries. The libraries were sequenced on the Illumina HiSeq 2500 platform, and sequenced reads were processed using the CellRanger toolkit (version 2.1.0). The human and mouse genomes were mapped to the GRCh38 human genome reference and mm10 mouse genome reference, respectively, using STAR [36].

### scRNA-seq data processing and identification of cell types

Gene expression matrices were generated using the Seurat R package (version 2.1) [37]. Cells with more than 150,000 or fewer than 1000 unique molecular identifiers (UMIs), more than 10,000 or fewer than 500 expressed genes, an UMI proportion over 10% for the mitochondrial genome, or an average expression level of two housekeeping genes (beta-actin and glyceraldehyde 3-phosphate dehydrogenase) below 3 were excluded. Additional cutoff criteria were applied to PDX samples to remove human–mouse mixed Gems. The human (mouse) cells containing at least 1% of mouse (human) reads were considered multiplets and removed. Because tumor cells had more expressed genes than non-tumor cells, strict criteria (over 2000 expressed genes) were applied to tumor cell clusters (as described as follows). Thereafter, outliers of each subtype were excluded from non-tumor cell clusters. Finally, undetermined cells from inferred CNV results were also discarded in order to identify the characteristics of pure cell types. After removing all unreliable cells, 2075 primary cells, 82 PDX human cells, and 958 PDX mouse TME cells were taken forward for further analyses.

In order to remove low-expression genes and detect rare cell types, genes expressed in 1% or more of the cells were used for downstream analyses. The data were transformed to TPM-like values by normalizing for differences in coverages and sequencing depth, and the TPM-like values were log^2^-transformed after adding a value of one. Variable genes were selected based on the average expression and dispersion (variance/mean) ratio for each gene, and principal component (PC) analysis was performed. In total, 20 significant PCs were selected based on the jackStraw function and elbow plot in R and used for graph-based clustering and t-distributed stochastic neighbor embedding visualization. Subsequently, each cluster was annotated with the average expression levels of known marker genes of a specific cell type. The relationship between the CNV score and CNV correlation was used to identify tumor cells. Differentially expressed genes of each cluster or subgroup were extracted using Student’s *t* test (Seurat package with default parameters).

### Pathway analysis

Gene set variation analysis (GSVA R package with RNA-seq mode) was used to estimate the activation levels of biological pathways and signatures using the “Canonical pathway” and the “Hallmark gene sets” obtained from the MsigDB website (http://software.broadinstitute.org/gsea/msigdb) and the cell cycle-related gene set [38].

### Cell cycle analysis

GSVA enrichment scores for the G1/S and G2/M phases were used to determine the cell cycle status of each cell. Cells were classified into cycling (both scores > 0), non-cycling (both scores < 0), and intermediate (one of scores > 0) using an empirical criterion.

### Analysis of receptor–ligand interaction

For putative receptor–ligand pairing analysis, we used immune checkpoint inhibitor (ICI) candidate interactions and curated human ligand–receptor pairs [39]. We used all expressed genes and considered receptor–ligand pairs by linking one cell expressing a receptor gene to another expressing a ligand gene. Thereafter, we summed the number of pairs by cell type or cell subtype and built an interaction network plot using igraph [40] and circlize [41] R packages. The size of the nodes and lines were scaled to 1/20 and 1/4,000,000 levels, respectively.

### Statistical analysis

For experimental data, all values are expressed as the mean ± standard deviation or standard error of the mean. Comparisons between two groups were performed using Student’s *t* test. One-way analysis of variance was applied for comparisons between more than two groups and to determine statistical significance for the fitting model of linear regression of two components. All *P* values are two sided, and *P* < 0.05 was considered statistically significant. All data analyses were performed using the SPSS statistical software, version 19.0 (SPSS, Inc., Chicago, IL, USA). For computational data, the chi-squared test was used to identify the cellular composition changes between the two groups.

## Results

### Elucidation of targetable oncogenic drivers in a case of chemo-refractory metastatic MIUBC

**Fig. 1:**
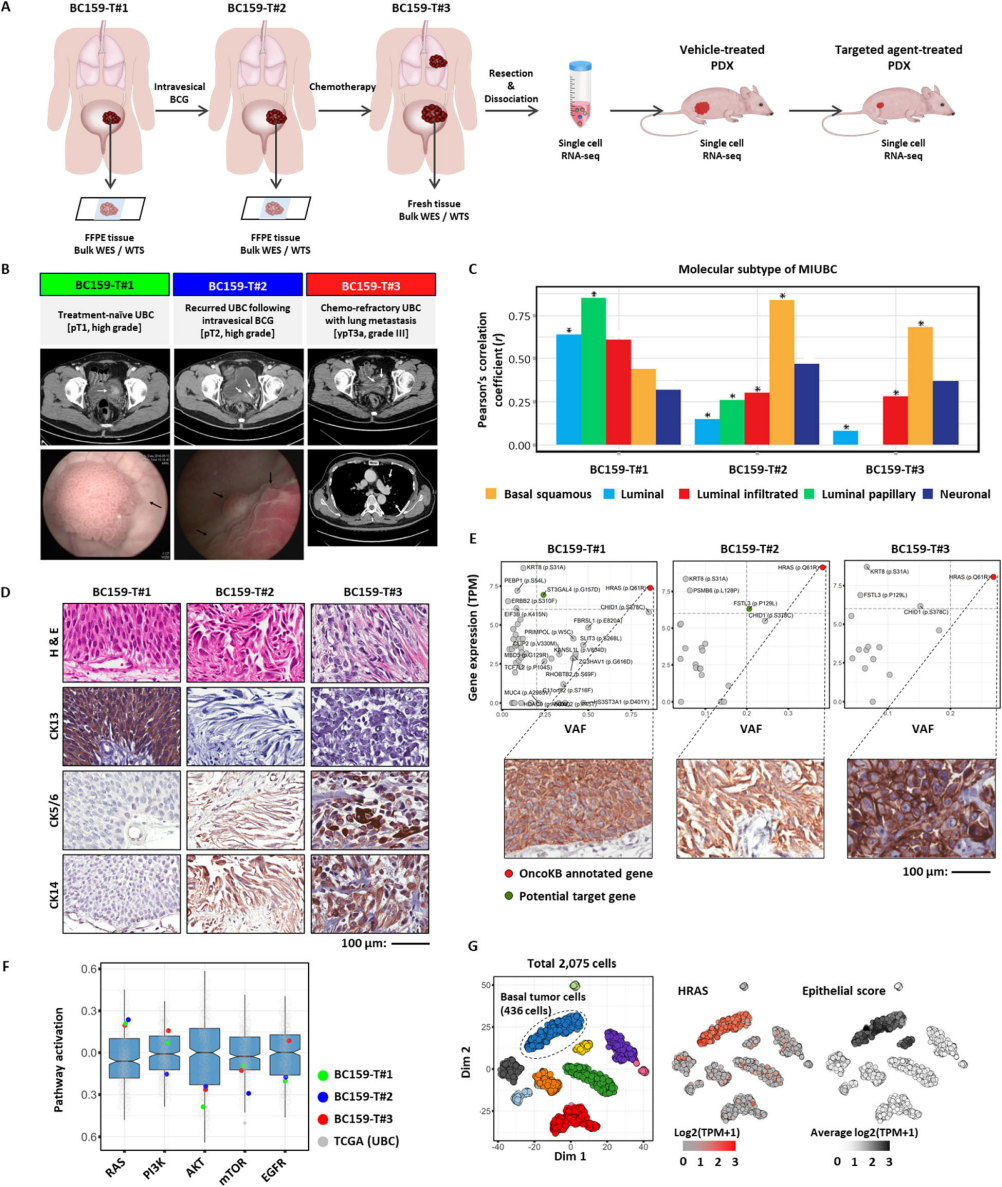
Genomic characteristics and the identification of a druggable target of a chemo-refractory metastatic muscle-invasive urothelial bladder cancer (MIUBC). **a** Overall workflow. Abbreviations: BCG, Bacillus Calmette–Guerin; WES, whole exome sequencing; WTS, whole transcriptome sequencing. **b** Clinical course of the patient investigated in the present study. **c** The mRNA expression-based molecular subtype of MIUBC identified by Pearson’s correlation coefficients between the bulk RNA sequencing samples and pre-defined Cancer Genome Atlas Urothelial Bladder Carcinoma (TCGA-BLCA) samples. *P < 0.05. **d** Immunohistochemistry staining of cytokeratin (CK)13, CD5/6, and CK14 performed on tumor sections to validate the molecular subtype of each sample. Abbreviation: H&E, hematoxylin and eosin. Scale bar, 100 μm. **e** Scatter plots showing mRNA expression levels versus variant allele frequencies (VAFs) for nonsynonymous mutation genes. Gray lines indicate cutoff of potential treatment target (VAF > 0.2 and gene expression > 6). Potential treatment target genes are marked as colored dots. Immunohistochemistry staining of HRASQ61R (insets) demonstrates protein expression. Scale bar, 100 μm. **f** The activation scores of core cancer-related pathways are plotted for BC159-T#1, #2, #3, and TCGA-BLCA samples. The RAS pathway was activated in all BC159-T samples compared with TCGA-BLCA samples. Each box shows the median and IQR (interquartile range, 25th to 75th percentiles), and whiskers indicate the highest and lowest value within 1.5 times the IQR. **g** The in-depth validation at single-cell level. T-distributed stochastic neighbor embedding (tSNE) plot of 2075 cells in BC159-T#3 sample, color-coded by their graphic-based clusters; basal tumor cell cluster is marked as a circle (left panel). HRAS gene is overexpressed in basal tumor cells (middle panel). Epithelial cells were identified by the average expression of epithelial-related genes (right panel)

An analytical scheme following the clinical course of a 49-year-old male patient is summarized in Fig. 1a and b. The patient was initially diagnosed with non-MIUBC (pT1, high grade) at the first transurethral resection of the bladder tumor (TUR-BT) (BC159-T#1), which progressed into MIUBC (pT2, high grade) at the second TUR-BT (BC159-T#2), despite intravesical Bacillus Calmette–Guerin (BCG) instillation. After undergoing multiple neoadjuvant chemotherapy regimens (gemcitabine/paclitaxel and sequential paclitaxel), the patient developed local recurrence and multiple lung metastases indicative of platinum-refractory metastatic MIUBC and for which effective systemic therapeutic options were limited [42, 43]. To reduce the tumor burden and relieve urological symptoms, the left ureterovesical junction of the recurred tumor (BC159-T#3) was removed following palliative partial cystectomy (ypT3aN1) (Fig. 1b). The molecular subtypes of these serial three tumors (BC159-T#1~#3) were determined by WTS (Fig. 1c) and IHC (Fig. 1d). BC159-T#1 was enriched in the luminal–papillary subtype-like gene signature, with CK13 expression, whereas BC159-T#2 and BC159-T#3 were classified as basal/squamous subtypes (CK5/6+ and CK14+) [32–34]. This observation was consistent with a well-known evolutionary path for MIUBC progression, from the luminal subtype to the basal squamous subtype, which demonstrates enhanced clinical aggressiveness through increased stemness and an epithelial-to-mesenchymal transition (EMT) [32–34].

To identify an oncogene addiction and ubiquitous truncal mutations on the tumor evolutionary tree, comprehensive WES of BC159-T#1~#3 was performed and a variety of single-nucleotide variations (SNVs; Additional file 1: Table S1) and CNVs (Additional file 1: Table S2) were attained. In addition, drugs targeting these genetic variations were annotated using the OncoKB (https://oncokb.org) [44] (Additional file 1: Table S1 and S2). Remarkably, an activating missense mutation in *HRAS* (c.182A>G; HRASQ61R) [45, 46] was found at the highest variant allele frequency (VAF) in all three tumors (Fig. 1e; Additional file 1: Table S1). The mutation was accompanied by tumor cell-specific overexpression of the mutant HRASQ61R protein (Fig. 1e, insets) and the RAS pathway activation compared with other cases of TCGA-BLCA (the top 7.5%) (Fig. 1f). scRNA-seq of BC159-T#3 (Additional file 2: Figure S1 and S2) further confirmed the specific transcriptional upregulation of *HRAS* in tumor cells with basal squamous subtype marker expression, compared with low expression in non-malignant stromal and immune cells (Fig. 1g), indicating a strong dependence on HRASQ61R as a major truncal alteration driving tumor evolution in this advanced refractory case.

### Preclinical validation of the therapeutic potency of blocking HRASQ61R activation and its clinical application

**Fig. 2:**
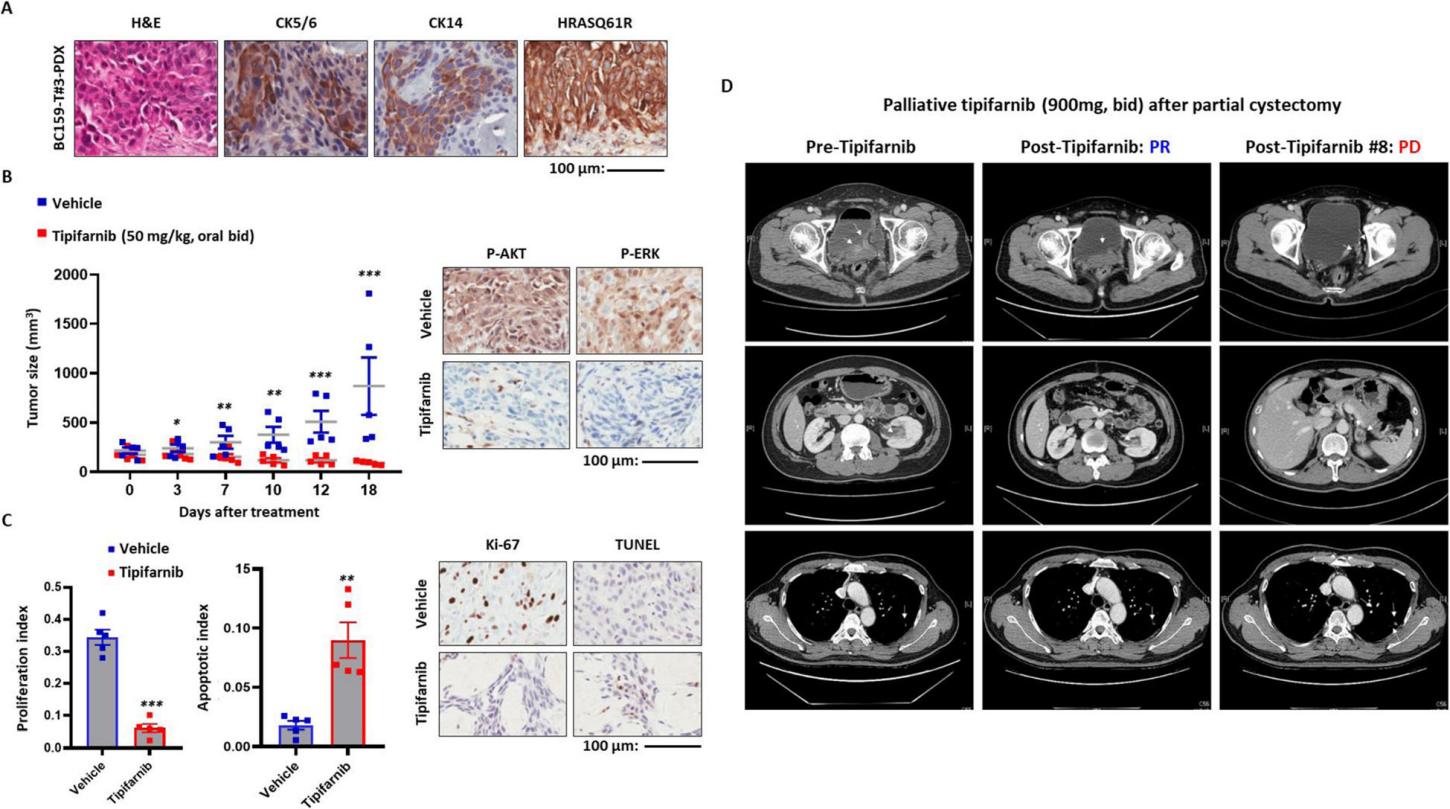
In vivo and clinical efficacy of the HRAS-targeting treatment. **a** Immunohistochemistry staining of protein markers of basal squamous subtype (CD5/6, CD14) and mutant HRASQ61R protein performed on tumor sections from patient-derived xenograft (PDX) of BC159-T#3. Abbreviation: H&E, hematoxylin and eosin. Scale bar, 100 μm. **b** Tumor sizes were measured in BC159-T#3 PDX administered tipifarnib (50 mg/kg) or vehicle as control. ***P < 0.001, **P < 0.01, *P < 0.05, Error bars indicate standard deviation (SD) (left panel). Immunohistochemistry staining of phosphorylated-AKT (P-AKT) and ERK (P-ERK) using PDX to evaluate the inhibitory effects of tipifarnib on HRAS downstream pathways (right panel). **c** Comparison of tumor cell proliferation and apoptosis between the tipifarnib and vehicle groups. ***P < 0.001, **P < 0.01, *P < 0.05. Error bars indicate SD (left panel). Immunohistochemistry staining of protein markers of proliferation (Ki-67) and apoptosis (TUNEL) performed on PDX tumor sections to validate the therapeutic efficacy (right panel). **d** Clinical responses to tipifarnib of the patient evaluated by serial chest–abdomen–pelvis computed tomography (CT). Left panel, before initiation of tipifarnib; middle panel, partial response to tipifarnib; right, the progression of primary tumor and lung metastases due to the resistance to tipifarnib

To test our hypothesis that therapeutic targeting of oncogenic HRAS could be a clinically relevant choice for the patient, we used subcutaneous BC159-T#3 PDX, which recapitulated the patient tumor with respect to tumor cell morphology, the expression of HRASQ61R protein, and basal squamous subtype (Fig. 2a). Tipifarnib is a highly potent and selective farnesyltransferase inhibitor (FTI) that can be particularly effective against advanced MIUBCs harboring mutated *HRAS* by dramatically attenuating farnesylation-mediated HRAS signaling [47–49]. When HRAS activation was pharmacologically inhibited by tipifarnib (50 mg/kg, oral, twice a day), tumor growth was significantly suppressed (Fig. 2b, left panel; Additional file 2: Figure S3) and HRAS-mediated ERK and AKT activation was reduced (Fig. 2b, right panel). Moreover, a decrease in cell proliferation and an increase in apoptosis supported its therapeutic efficacy (Fig. 2c). On the basis of the promising preclinical anti-tumor efficacy of tipifarnib, the patient was enrolled in a tipifarnib phase II trial for pre-treated, heavily treated, metastatic urothelial carcinomas harboring mutated HRAS (ClinicalTrials.gov, NCT02535650, palliative, 900 mg, bid) following a partial cystectomy, and the patient showed an obvious decrease in tumor burden in the bladder and left renal calyx, as well as in lung metastases (partial response) (Fig. 2d, middle panels). Unfortunately, consistent with our findings that tipifarnib administration failed to achieve complete remission of BC159-T#3 PDX, the dissipating tumor started to regrow in the patient after 9 months (Fig. 2d, right panels).

### Elucidation of tumor cell-intrinsic factors for resistance to HRAS-targeted monotherapy by scRNA-seq

**Fig. 3:**
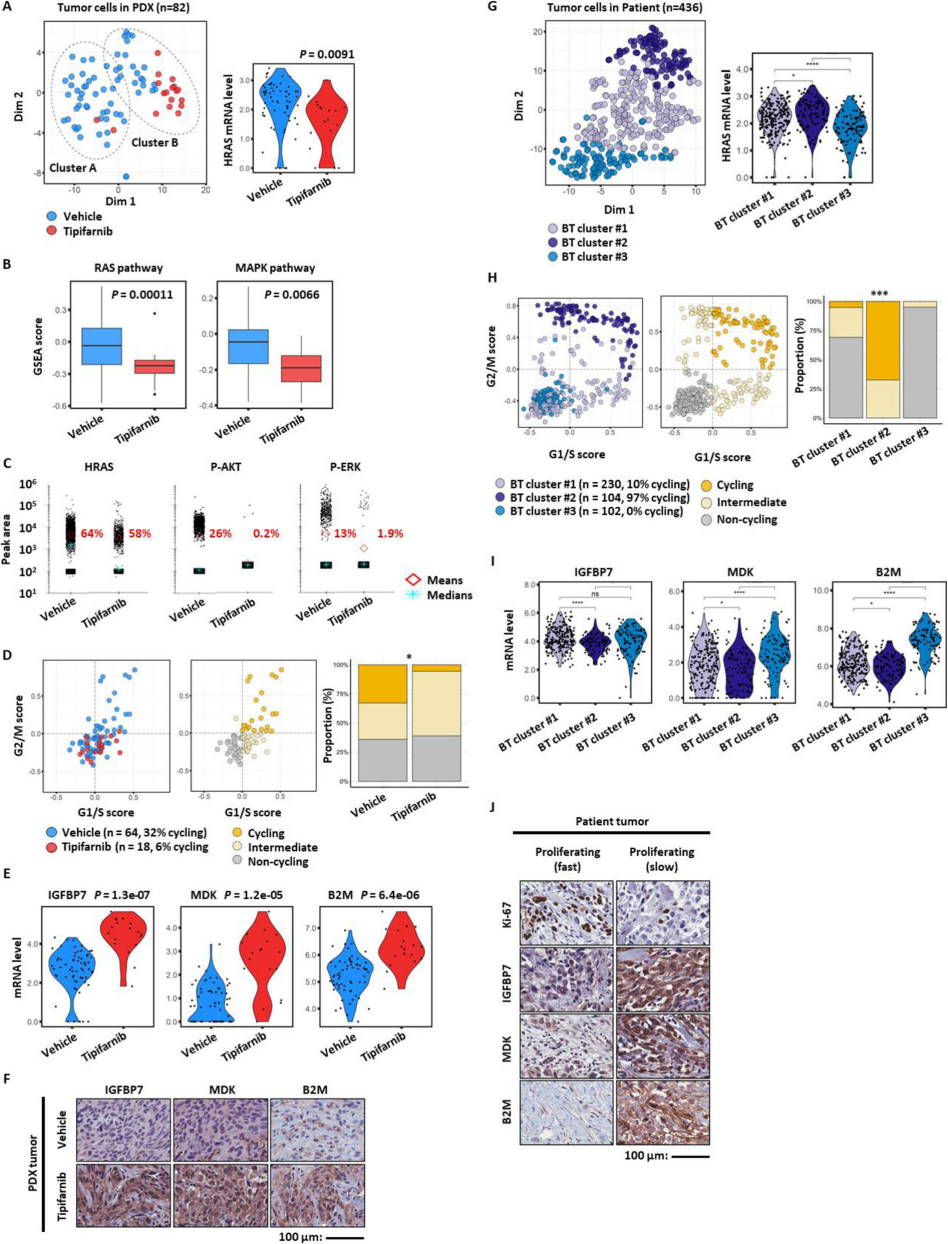
Tumor cell-intrinsic factors underlying treatment resistance to tipifarnib. **a** tSNE plot of total human tumor cells (dots) from the PDX model of BC159-T#3, color-coded by sample origin, circled by cluster (left panel). Comparison of *HRAS* mRNA expression level in tumor cells treated with tipifarnib or vehicle as control. Dots indicate individual cells, *P* = 0.0091 (right panel). **b** Comparison of RAS and MAPK pathway activity in tumor cells treated by tipifarnib and vehicle as control. *P* = 0.00011, *P* = 0.0066, respectively. Each box shows the median and IQR (interquartile range, 25th to 75th percentiles), whiskers indicate the highest and lowest value within 1.5 times the IQR, and outliers are marked as dots. **c** Single-cell western blots of HRAS protein and its downstream protein markers (p-AKT and p-ERK) in tumor cells treated with tipifarnib or vehicle. Dots indicate individual cells, and diamond and star shapes indicate means and median of peak area, respectively. **d** Prediction of cell cycle state in tumor cells from PDX at a single-cell level (dots) using G1/S and G2/M module score (left panel). Cells are colored by their assigned cell cycle state (cycling; orange, intermediate; yellow, non-cycling; gray, middle panel). The relative cellular composition for tumor cells treated with tipifarnib or vehicle. Chi-squared test, *P* = 0.021, *P < 0.05 (right panel). **e** Violin plots of significantly upregulated genes, *IGFBP7*, *MDK*, and *B2M*, in tumor cells treated by tipifarnib or vehicle (dots). *P* = 1.3e−07, *P* = 1.2e−05, *P* = 6.4e−06, respectively. **f** Validation of comparative upregulation of IGFBP7, MDK, and B2M in tumor cells treated with tipifarnib compared to those with vehicle by immunohistochemistry staining performed on PDX. Scale bar, 100 μm. **g** tSNE plot of total tumor cells from BC159-T#3 patient tumor, color-coded by cluster (left panel). Violin plot of HRAS mRNA expression level against distinct tumor cell clusters of BC159-T#3. Student’s *t* test, ****P < 0.0001, *P < 0.05. Abbreviation: BT, basal tumor (right panel). **h** Prediction of cell cycle state in tumor cells from BC159-T#3 at a single-cell level (dots) using G1/S and G2/M module score (left panel). Cells are colored by cell cycle as in **d** (middle panel). The relative cellular composition among basal tumor clusters. Chi-squared test, *P* < 2.2e−16, ****P < 0.0001 (right panel). **i** Violin plots of mRNA expression levels of *IGFBP7*, *MDK*, and *B2M* against distinct tumor cell clusters of BC159-T#3 (dots). ****P < 0.0001, *P < 0.05. **j** Comparison of IGFBP7, MDK, and B2M protein expression between fast (upregulated Ki-67) and slow (downregulated Ki-67) growing regions in BC159-T#3 by immunohistochemistry staining. Scale bar, 100 μm

In the current study, the PDX tumors that endured tipifarnib treatment (residual tumors in tipifarnib-treated BC159-T#3 PDX) would enable us to analyze tumor cells as well as the TME that allowed survival and the emergence of treatment resistance. In previous studies, scRNA-seq could identify distinct gene modules expressed across residual tumor cells and a variety of tumor-associated non-malignant cells within a surrounding activated stroma upon treatment, which have accelerated the refractoriness of tumors [6, 7, 11, 13]. Subsequently, we used scRNA-seq to perform pairwise comparisons of tipifarnib-treated and untreated PDX tumors (Figs. 3a~f and 4; Additional file 2: Figure S4) to delineate the mechanisms of resistance originating from tumor-intrinsic and/or TME-mediated activation of salvage pathways other than HRAS.

**Fig. 4:**
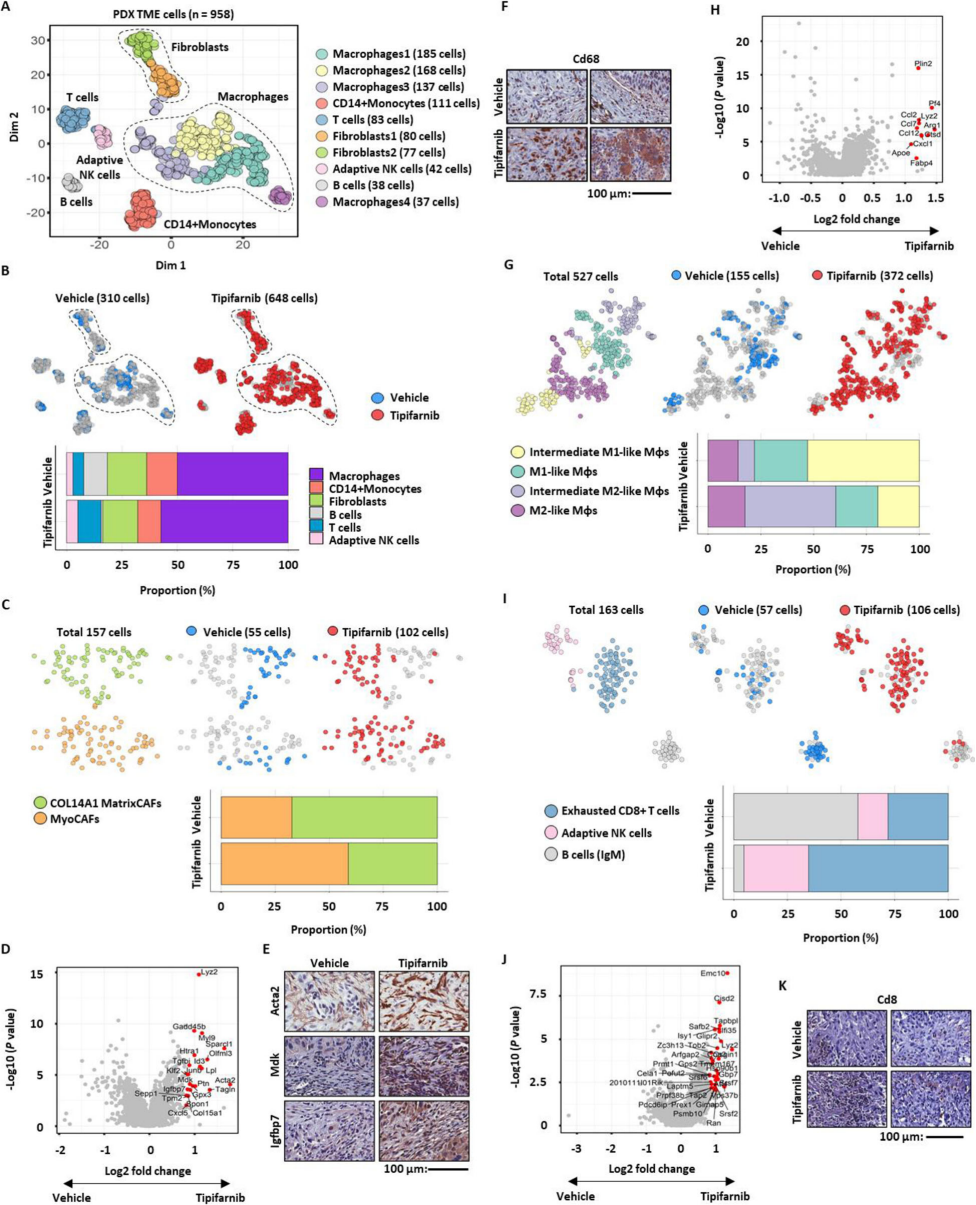
Tumor cell extrinsic factors underlying treatment resistance to tipifarnib. **a** tSNE plot of non-tumor mouse cells (dots) from PDX of BC159-T#3, color-coded by cluster, circled by global cell type. **b** tSNE plot, color-coded by sample origin and circled by cell type (top panel). Relative cellular composition of non-tumor mouse cells treated with tipifarnib or vehicle (bottom panel). **c** tSNE plot (top panel) and relative cellular composition (bottom panel) of fibroblasts from PDX mouse cells treated with tipifarnib or vehicle, color-coded by cluster (top left panel) or sample origin (top middle and right panel). **d** Volcano plot of differentially expressed genes in mouse fibroblasts from PDX treated with tipifarnib or vehicle. Genes with fold change > 0.8 with *P* < 0.001 are colored in red. **e** Validation of relative overexpression of Igfbp7 and Mdk in mouse cancer-associated myofibroblasts (MyoCAFs, ACTA2 positive) from the tipifarnib group compared to those from the vehicle group by immunohistochemistry staining. Scale bar, 100 μm. **f** Increased infiltration of mouse macrophages (CD68 positive) in PDXs treated with tipifarnib confirmed by immunohistochemistry staining. Scale bar, 100 μm. **g** tSNE plot (top panel) and relative cellular composition (bottom panel) of mouse macrophages from PDX treated with tipifarnib or vehicle, color-coded by cluster (top left panel) and sample origin (top middle and right panel). **h** Volcano plot of differentially expressed genes between vehicle and tipifarnib-treated mouse macrophages from PDX. Genes with fold change > 0.8 with *P* < 0.001 are colored in red. **i** tSNE plot (top panel) and relative cellular composition (bottom panel) of mouse lymphocytes from PDX treated with tipifarnib or vehicle, color-coded by cluster (top left panel) and sample origin (top middle and right panel). **j** Volcano plot of differentially expressed genes between vehicle and tipifarnib-treated mouse T cells from PDX. Genes with fold change > 0.8 with *P* < 0.001 are colored in red. **k** Increased infiltration of mouse T cells (CD8 positive) in PDX treated with tipifarnib confirmed by immunohistochemistry staining. Scale bar, 100 μm

Cancer cells display significant plasticity, which influences how they respond to treatments [2, 3]. Tumor cells in the PDX that survived tipifarnib treatment showed a significant downregulation of *HRAS* as well as RAS and MAPK signaling-related genes (Fig. 3a, b). Suppressed HRAS-mediated signaling by tipifarnib in each tumor cell was also confirmed at the protein level by single-cell Western blotting (Fig. 3c). Most interestingly, the tipifarnib-resistant tumor cells were in a state of cell dormancy [50], which was characterized by an increased proportion of non-cycling cells (Fig. 3d), contributing to resistance to agents targeting cell proliferation. Furthermore, the relative increase in the transcriptional and translational expression level of insulin-like growth factor-binding protein 7 (*IGFBP7*), midkine (*MDK*), and beta-2-microglobulin (*B2M*) genes was observed in remnant tumor cells after tipifarnib treatment (Fig. 3e, f).

Furthermore, unsupervised clustering analysis of all PDX tumor cells from two groups identified two distinct clusters (A and B; Fig. 3a; Additional file 2: Figure S5). The signaling pathways where gene expression was significantly different between clusters A and B are summarized in Additional file 2: Figure S5B~D. The tumor cells in cluster B showed similar characteristics to those of tipifarnib-resistant tumor cells, including downregulation of the RAS and MAPK signaling pathways (Additional file 2: Figure S5C) and upregulation of *IGFBP7*, *MDK*, and *B2M* mRNA (Additional file 2: Figure S5D, E). Notably, a few cells in cluster B belonged to the control group (Fig. 3a, left panel), indicating the existence of putative tipifarnib-resistant tumor cells before treatment initiation.

To confirm the existence of a matched subpopulation with intrinsic resistance to tipifarnib in the patient sample, we explored the scRNA-seq data for the resected BC159-T#3 before tipifarnib treatment (Fig. 3g–j). In the patient sample, three distinct subgroups (basal tumor clusters #1, #2, and #3; Fig. 3g, left panel) were identified by unsupervised clustering of tumor cells. The basal tumor cluster #3 demonstrated transcriptional downregulation of *HRAS* (Fig. 3g, right panel); cell dormancy (Fig. 3h); and a higher expression of *IGFBP7*, *MDK*, and *B2M* (Fig. 3i), recapitulating those of the tipifarnib-resistant (or cluster B) BC159-T#3 PDX tumor cells. In conjunction with these data, IHC analysis of the patient tumor revealed that Ki-67-negative, non-cycling cells showed a higher immunoreactivity against IGFBP7, MDK, and B2M (Fig. 3j). To summarize, downregulation of the drug target, cell dormancy, and upregulation of the IGFBP7, MDK, and B2M that were common characteristics of tumor cells in patient basal tumor cluster #3 and PDX cluster B could contribute to therapeutic resistance to tipifarnib, indicating a selection for intrinsically resistant basal tumor cluster #3 subpopulations in BC159-T#3. For example, B2M could induce resistance to tipifarnib by inducing tumor cell survival, aggressiveness, and EMT via the induction of RAS-independent activation of the phosphoinositide 3-kinase (PI3K)/AKT/mammalian target of rapamycin (mTOR) and ERK signaling pathways [51, 52].

### TME contribution to acquired resistance to tipifarnib

Treatment-induced alterations in the TME also generate a protective niche, or a shielding reservoir, for cancer cells, which in turn facilitates tumor relapse and progression [6, 7]. In order to elucidate the TME factors that induce resistance against tipifarnib, we analyzed scRNA-seq data of mouse TME cells in the tipifarnib-treated and control PDX groups (Additional file 2: Figure S4). Unsupervised clustering revealed the presence of murine TME components in the PDX, including fibroblasts, monocytes/macrophages, T and B cells, and natural killer (NK) cells (Fig. 4a; Additional file 2: Figure S6). Although tipifarnib induced minor changes in the global cell composition (Fig. 4b), detailed analysis of each cell component unveiled alterations in specific cell subtypes after tipifarnib treatment.

First, collagen alpha-1(XIV) chain (COL14A1)-expressing matrix cancer-associated fibroblasts (CAFs) (COL14A1 MatrixCAFs) [53] and actin alpha 2, smooth muscle (ACTA2)-expressing cancer-associated myofibroblasts (MyoCAFs) [54, 55] were identified within the fibroblast cluster in vehicle- and tipifarnib-treated PDX (Fig. 4c; Additional file 2: Figure S7). Notably, tipifarnib treatment increased the number of MyoCAFs, which are often associated with tumor aggressiveness and a dismal prognosis [54, 55], whereas the control group contained more COL14A1 MatrixCAFs (Fig. 4c). The tipifarnib-induced dynamics of the fibroblast cluster were reflected by differential gene expression between control and tipifarnib-treated fibroblasts (Fig. 4d; Additional file 1: Table S3; Additional file 2: Figure S7). Interestingly, *Mdk* and *Igfbp7*, the tumor-intrinsic factors associated with resistance to tipifarnib (Fig. 3e, f), were also upregulated in MyoCAFs in the tipifarnib-treated group (Fig. 4d, e), implying key roles for these two targets in dormant tumor cells. Thus, the MyoCAF subset crosstalk may contribute to resistance to tipifarnib, whereas the pre-existing tipifarnib-resistant subpopulation may survive tipifarnib treatment.

Second, tipifarnib treatment resulted in a marked increase in the total number and proportion of macrophages (Fig. 4b, f), accompanied by a decrease in M1-like macrophages but an increase in M2-like macrophages [56, 57] within the macrophage pool (Fig. 4f, g). Consequently, macrophages in the treated group showed higher expression of arginase 1 (*Arg1*), C-C motif chemokine ligand 2/7/12 (*Ccl2*, *Ccl7*, and *Ccl12*), chemokine (C-X-C motif) ligand 1 (*Cxcl1*), and apolipoprotein E (*Apoe*) associated with metabolic and chemotactic mediators in M2 macrophages [56–58] than those in the control group (Fig. 4g, h; Additional file 1: Table S4; Additional file 2: Figure S8). Despite the compromised adaptive immune system in nude mice, recent work has shown the presence of functional T cells in the BALB/c nude mouse spleen [59]. Interestingly, we recovered a significant number of exhausted CD8^+^ cytotoxic T lymphocytes (CTLs) [60, 61] (Additional file 1: Table S5) and adaptive NK cells [62, 63] in the treated group and a small number of B cells, expressing IgM, in the control mice (Fig. 4i; Additional file 2: Figure S6 and S9). Overall, the tipifarnib-induced dynamics of TME cells were characterized by an increased infiltration of tumor-promoting MyoCAFs and suppressive immune cells, such as M2 macrophages and exhausted CTLs.

**Fig. 5:**
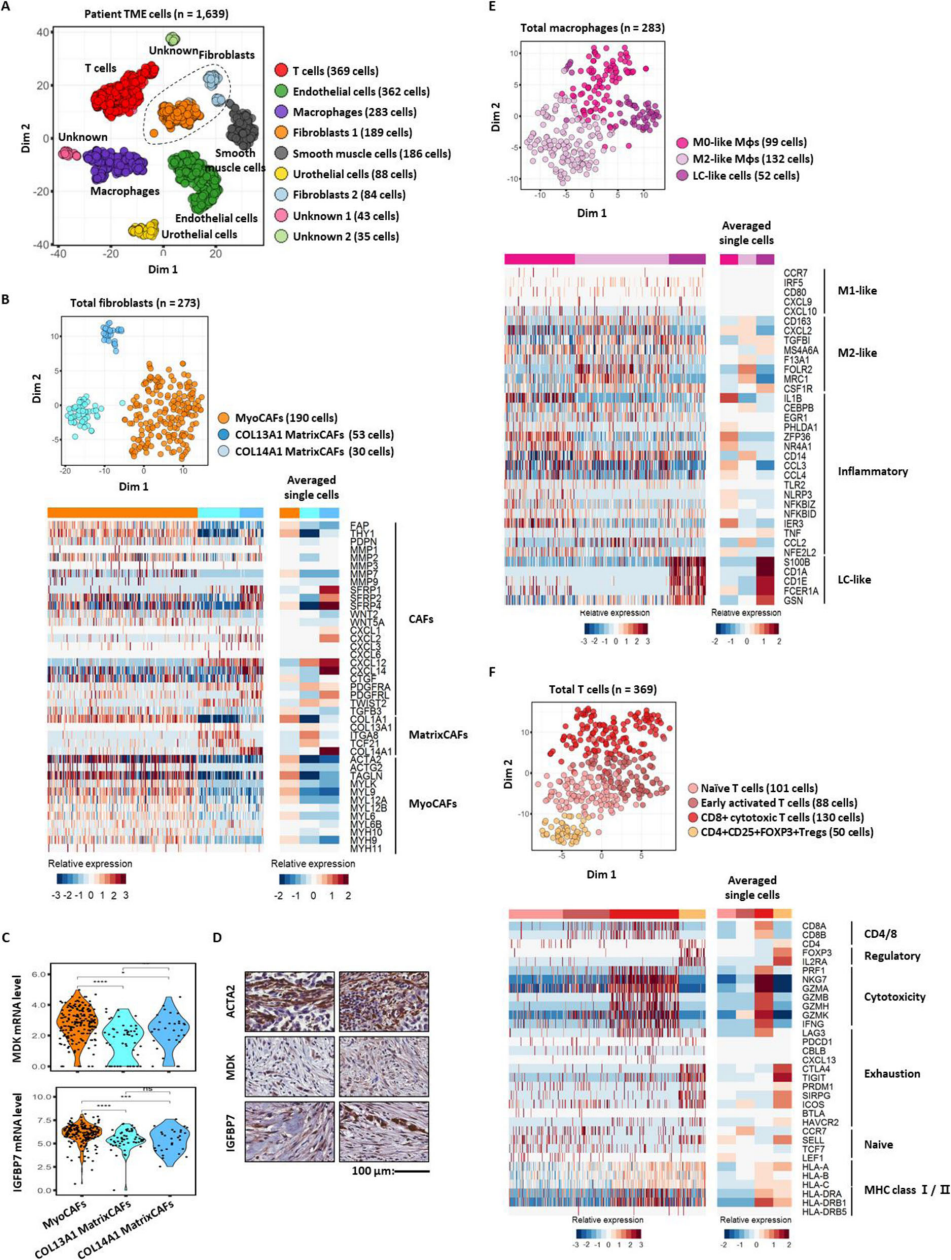
Parallel analysis of the tumor microenvironment in BC159-T#3. **a** tSNE plot of non-tumor cells (dots) from BC159-T#3 sample, color-coded by cluster; fibroblasts are circled. **b** tSNE plot of human fibroblasts (dots), color-coded by cluster (top panel). Heatmaps of single cells (bottom left panel) and averaged single cells (bottom right panel) represent the mRNA expression levels of well-known markers for fibroblasts. **c** Violin plots of *MDK* and *IGFBP7* mRNA expression levels in three fibroblast clusters. Abbreviations: MyoCAFs, cancer-associated myofibroblasts; COL13A1 MatrixCAFs, COL13A1 expressing cancer-associated matrix fibroblasts; COL14A1 MatrixCAFs, COL14A1 expressing cancer-associated matrix fibroblasts. ****P < 0.0001, ***P < 0.001, **P < 0.01, *P < 0.05. **d** Validation of protein expression of IGFBP7 and MDK in fibroblasts (ACTA2 positive) from BC159-T#3 by immunohistochemistry staining. Scale bar, 100 μm. **e** tSNE plot of human macrophages (dots), color-coded by cluster (top panel). Heatmaps of single cells (bottom left panel) and averaged single cells (bottom right panel) represent the mRNA expression levels of well-known markers for macrophage subtypes. Abbreviation: LC-like cells, Langerhans cell-like cells. **f** tSNE plot of human T cells (dots), color-coded by cluster (top panel). Heatmaps of single cells (bottom left panel) and averaged single cells (bottom right panel) represent the mRNA expression levels of well-known markers of T cell subtypes

A parallel analysis of the patient TME for BC159-T#3 (Fig. 5a; Additional file 2: Figure S1) identified diverse cellular components comparable with those of the murine TME, including fibroblasts, monocytes/macrophages, and T cells. Within the fibroblast cell types, we found COL13A1- (COL13A1 MatrixCAFs) and COL14A1-type matrix CAFs (COL14A1 MatrixCAFs), as well as MyoCAFs (Fig. 5b; compared with the murine TME in Additional file 2: Figure S7). Similar to those from the murine TME, BC159-T#3-infiltrating MyoCAFs specifically expressed higher levels of *MDK* and *IGFBP7* than other matrix fibroblasts (Fig. 5c, d). Among the monocytes and macrophages, we found M0-type, M2-type, and Langerhans cell-like subpopulations (Fig. 5e), which are tissue-specific macrophage subsets that share typical features with dendritic cells in terms of their migratory potential and ability to stimulate T cells; this was reflective of the history of intravesical BCG therapy in this patient [64, 65] (Fig. 5e). Most importantly, we also demonstrated the infiltration of heterogeneous CD4^+^ and CD8^+^ T cells in dynamic states, ranging from naïve to activated CTLs as well as CD4^+^CD25^+^forkhead box P3 (*FOXP3*)^+^ regulatory T cells (Tregs) in BC159-T#3 (Fig. 5f, top panel). Both activated CTLs and Tregs expressed varying levels of immune checkpoint genes, such as cytotoxic T lymphocyte-associated protein 4 (*CTLA4*), lymphocyte activation gene-3 (*LAG3*), programmed cell death protein 1 (*PDCD1*), and T cell immunoreceptor with Ig and ITIM domains (*TIGIT*) [66–68] (Fig. 5f, bottom panel).

Taken together, PDX could recapitulate the diverse cell types present in the human TME, thus providing a unique opportunity to monitor alterations inflicted by tumor cell-targeted tipifarnib treatment. These TME cell components and characteristics, together with the post-tipifarnib PDX data, suggest that the inhibition of myofibroblasts and anti-inflammatory macrophages, or the reinvigoration of T cell immunity, could be considered as therapeutic tactics for the tipifarnib-resistant MIUBC.

### Personalized salvage regimen against tumor relapse after tipifarnib treatment

**Fig. 6:**
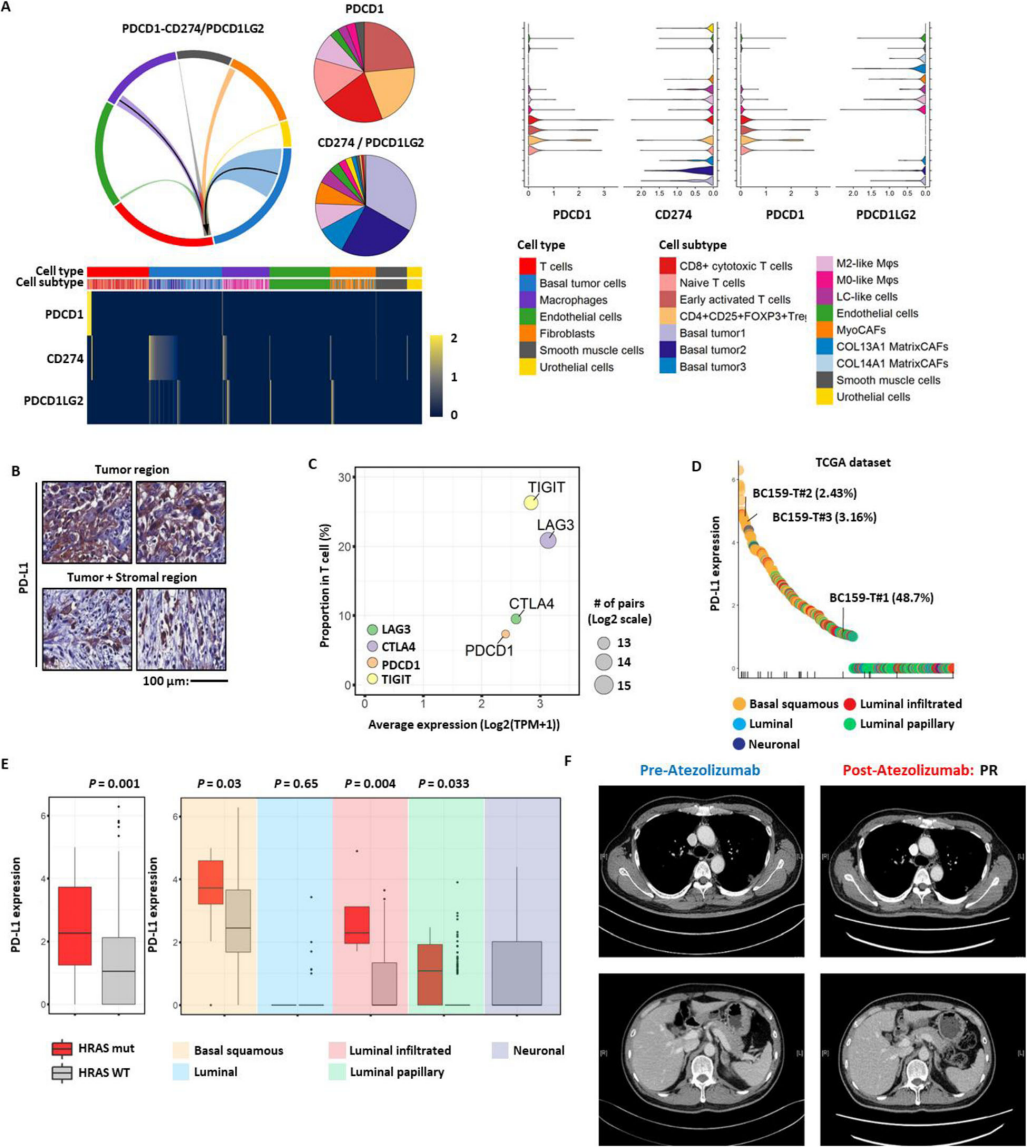
Programmed death receptor 1/programmed death-ligand 1 inhibitor as a potential treatment strategy against tipifarnib-resistant tumors developed from BC159-T#3. **a** Receptor–ligand interaction between the programmed death receptor 1 (PD-1) and its ligands (PD-L1/PD-L2) of total cells from BC159-T#3. Arrows indicate the direction of interaction (from ligand to receptor) that expresses more than 10% of the ligand genes (left top panel). Pie charts demonstrating the cell composition that express *PDCD1/PD-1*, *CD274/PD-L1*, and *PDCD1LG2/PD-L2* genes (left top panel). Heatmap of single cells showing the mRNA expression levels of *PDCD1/PD-1*, *CD274/PD-L1*, and *PDCD1LG2/PD-L2* genes (left bottom panel). 2D-violin plots represent each interaction of PD-1-PDL1 or PD-L2 (right panel). **b** Representative images for immunohistochemical staining of PD-L1 in BC159-T#3. Expression is specific in tumor cells in the core and stromal border. Scale bar, 100 μm. **c** Scatter plot representing the average expression of target ligands (*x*-axis) and the proportion of target receptors in T cells (*y*-axis). Circle size is proportional to the number of pairing between target ligands and receptors in log_2_ scale. **d***CD274/PD-L1* mRNA expression levels are plotted for BC159-T#1, #2, #3, and TCGA-BLCA samples. All samples are colored by molecular subtype. The samples with HRASQ61R mutation are marked in the bottom row of vertical ticks. **e***CD274/PD-L1* mRNA expression levels according to HRASQ61R mutation. Patients with HRASQ61R mutation in basal squamous subtype show upregulated expression of *CD274/PD-L1*. Each box shows the median and IQR (interquartile range, 25th to 75th percentiles), whiskers indicate the highest and lowest value within 1.5 times the IQR, and outliers are marked as dots. **f** Clinical partial response to atezolizumab (a fully humanized, engineered monoclonal antibody of IgG1 isotype against PD-L1) evaluated by CT scan

Currently, co-targeting of dormant tumor cells and MyoCAFs by therapeutic inhibition of MDK and IGFBP7 is not available. However, programmed cell death-1 (PD-1)/PD-L1 inhibitors approved for second-line treatment of advanced chemo-refractory MIUBC [69–71] could be effective based on the immunosuppressive TME characterized by the infiltration of exhausted CTLs and Tregs overexpressing immune checkpoint genes such as PD-1/PD-L1 in BC159-T#3. Interestingly, we found a cell communication network, through an inference of immune checkpoint ligand–receptor interactions, between tumor cells and a variety of stromal/immune cells in BC159-T#3 (Fig. 6a, b; Additional file 2: Figure S10A). Specifically, tumor cells expressed PD-L1 (encoded by *CD274*) and *PVR transcripts*, which could transmit inhibitory signals to PD-1 (encoded by *PDCD1* gene) and TIGIT on T cells, respectively [66–68] (Fig. 6b, c; Additional file 2: Figure S10A). Macrophages and endothelial cells were the main sources of major histocompatibility complex (MHC) class II, interacting with LAG3, whereas macrophages predominantly expressed *CD80* and *CD86* transcripts, interacting with *CTLA4* (Additional file 2: Figure S10A). Most importantly, PD-L1 expression in tumor cells of BC159-T#3 was at the top 3.16% of the cases recorded in TCGA-BLCA dataset (Fig. 6d). These results were corroborated by higher PD-L1 expression in the basal subtype compared with that in other subtypes, particularly those harboring *HRAS* mutations (Fig. 6e). By comparison, the CD8/CD4 ratio (associated with a high fraction of CTLs relative to Tregs) and cytolytic scores quantified by transcript levels of granzyme A and perforin 1 [69, 70, 72] were indistinct for BC159-T#3 or *HRAS* mutant UBCs (Additional file 2: Figure S10B). Since the high expression levels of PD-L1 were predictive of favorable clinical response to PD-1/PD-L1 inhibitors, the patient who failed tipifarnib treatment was started on a humanized monoclonal PD-L1 antibody, atezolizumab, that is effective in patients with platinum-resistant MIUBC [73, 74] as optimized next-line therapeutic options for the relapsed tumor after tipifarnib treatment. Subsequent imaging demonstrated a partial response to atezolizumab in our subject as expected (Fig. 6f).

## Discussion

Tumor cells can alter the drug target itself, or rewire cellular signaling so as to negate the effect of the targeted agent, posing a significant challenge to monotherapy by accelerating the growth of other clones [2, 3]. Furthermore, because of its heterogeneity and complexity, the TME should not be neglected when investigating the interactions between a growing tumor and its surrounding stroma or its role in facilitating the emergence of drug resistance [5–7, 12, 14, 15]. Here, we demonstrate the power of single-cell transcriptomics depicting tumor landscape for the identification of personalized treatment options for one refractory advanced cancer patient. scRNA-seq analysis of a parental tumor addicted to a specific oncogenic pathway before treatment with a specific oncogene-targeting agent, as well as non-treated PDX and one treated with the same drug, suggested an advanced personalized sequential treatment option against a chemo-refractory and rapidly evolving case of metastatic MIUBC.

There are a number of reasons for using the indicated case in this study. First, advanced chemo-resistant MIUBC remains a challenging disease with poor outcomes and a paradigm shift to targeting selected patients with tumor-specific genetic targets has not yet been established in MIUBC [17, 19]. Second, *HRAS* mutations are one of the major driver alterations in MIUBCs (5.1–20%) [75, 76], and there is a consistency in the type of HRAS mutations among different tumor samples obtained from the same patients with MIUBC [45], suggesting that the majority of recurrences in MIUBC are clonally related [46]. Although we could not obtain tumor samples from lung metastasis because of multiple patterns and the difficulty of biopsy from a metastasis site, 100% consistency with HRAS alterations during the clinical course, specifically overexpressed HRASQ61R, and activation of downstream effectors confirmed at the single tumor cell level strongly indicated a RASophathy, and we hypothesized that HRAS-targeting strategies could be a potential palliative adjuvant therapy for this patient. Finally, FTIs such as tipifarnib have been developed as anti-RAS therapeutic agents [47–49], and moreover, wild-type *TP53* tumors containing mutated *HRAS* similar to our case are the most sensitive to FTIs because HRAS is activated by only farnesylation, in contrast to KRAS and NRAS which can be geranylgeranylated as well [77]. However, as a single agent, tipifarnib appears to have modest clinical effects in tumors that are driven through oncogenic HRAS function; these are insufficient to induce long-term tumor inhibition because of the complexities of HRAS [47–49, 78, 79]. The rapidly accelerated fibrosarcoma/mitogen-activated protein kinase kinase (MEK)/ERK, PI3K/mTOR/AKT, Ral guanine nucleotide exchange factor/Ral, and T cell lymphoma invasion and metastasis 1/Rac initiated by HRAS-mediated signaling pathways have divergent and critical roles in tumor progression and evolution through crosstalk and feedback interactions between tumor cell–tumor cell or tumor cell–TME [78, 79]. Therefore, rationale-driven combination strategies in synergy with tipifarnib are required to reduce the need for protracted therapy.

Invigorated by acquired resistance to tipifarnib, we postulated that the regrowth could originate from tumor-intrinsic and TME-mediated activation of salvage pathways other than HRAS, and performed in-depth analysis of tumor cells and non-neoplastic stromal cells in TME, from BC159-T#3 and the PDX tumors used in the tipifarnib in vivo efficacy study, with the aid of scRNA-Seq. In particular, application of scRNA-seq to the remaining tumors persisting in tipifarnib-treated BC159-T#3 PDX provided details from the tumor cells that survived tipifarnib treatment and from the tumor promoting stroma. Profiling of the PDX transcriptome at the single-cell resolution level enabled us to dissect changes in inherently complex and heterogeneous cell populations in response to the HRAS-targeting tipifarnib treatment. Extensive scRNA-seq-based analysis of the PDX and parental BC159-T#3 strongly indicated overall changes in the transcriptome landscape of the putative tipifarnib-resistant human tumor cells and mouse TME cells suggestive of broad tumor evolution in stromal and immune cell components by the HRAS-targeting treatment. The results suggest that tumor cells with molecular characteristics similar to those that have survived after tipifarnib treatment (tipifarnib-resistant subpopulation) probably exist in the tumor before treatment as distinct subpopulation with intrinsic resistance to tipifarnib. Most importantly, tumor cells that survived through tipifarnib therapy were in a relatively dormant stage. Dormancy, a slow-cycling, persistent quiescent state, allows cells to survive in a hostile tumor microenvironment under treatment pressure such as RAS/RAF/MEK inhibitors, which serves as a likely reservoir for the emergence of fully resistant proliferative cells [80–82].

On the other hand, benign cells in TME niches actively modulate cancer cell response to a broad range of standard chemotherapies and targeted agents, and therefore, cancer-oriented therapeutics combined with TME-targeting treatments are likely to yield optimal clinical outcomes by overcoming treatment failure; however, most studies addressing this disregard the heterogeneity in TME cues and niches that will differentially influence tumor evolution. scRNA-seq analysis, which enables dissection of heterogeneous drug target pathways at cellular resolution, may have a significant clinical utility for the design of tailored combination therapy targeting both tumor cells and associated TME [12–16]. The transcriptome landscape of the putative tipifarnib-resistant human BC159-T#3 and PDX at the single-cell resolution level enabled us to dissect changes in inherently complex and heterogeneous cell populations in response to tipifarnib HRAS-targeting treatment.

We demonstrated high rates of infiltration of M2 tumor-associated macrophages (TAMs) and MyoCAFs in the original tumor (BC159-T#3) and tipifarnib-treated PDX, suggesting that CAF and TAM inhibition or reinvigoration of anti-tumor T cell immunity could be therapeutic strategies to combat tipifarnib-resistant MIUBC. CAFs play important roles in shaping an immunosuppressive, tumor-permissive TME by preferentially inducing the tumor-promoting function of TAMs, and the combined activities of heterogeneous CAFs and TAMs via reciprocal interaction significantly induce recruitment and pro-tumoral activation of both cell types, which in turn accelerates tumor progression [83, 84], indicating that therapy targeting both TAMs and CAFs, or targeting the cell–cell interaction between TAMs and CAFs, should improve anti-tumor therapeutic efficacy. Interestingly, the existence of a tumor cell subpopulation and MyoCAFs that express high levels of *IGFBP7* and *MDK* contributing to tipifarnib failure was unveiled by scRNA-seq in this study. IGFBP7 and MDK expressed by tumor cells and CAFs promote tumor progression and relapse by enhancing cancer stemness, EMT, extracellular matrix remodeling, and angiogenic activity [85–87]. Despite inhibition of IGFBP7 and MDK may be a useful adjunct to co-targeting tumor cells and CAFs in patients with MyoCAF-rich tumors, few targeting agents are currently available for these molecules, which led us to focus on TME that showed immunosuppressive features. MAPK pathway inhibitors such as tipifarnib stimulate recruitment of immunosuppressive M2 TAMs, PD-L1 upregulation, induction of Treg, and suppression of anti-tumor responses of CTLs [88–90]. Our scRNA-seq data for PDX after HRAS-targeting treatment strongly indicate the involvement of an immunosuppressive TME, such as the enrichment of M2-type TAMs and exhausted CTLs. The scRNA-seq analysis of BC159-T#3 also showed the presence of M2-like macrophage subsets and infiltration of CTLs and Tregs. Furthermore, immune checkpoint-associated genes in these immune cells were significantly induced by tipifarnib treatment. Fortunately, the non-cycling dormant human tumor cells in BC159-T#3, also observed in tipifarnib-treated PDX, showed upregulation of antigen presenting molecules, such as class I MHC and B2M, which play a critical role in the presentation of tumor antigens for the recognition of tumor cells by CTLs [91]. Analysis of ligand–receptor interactions for the immune checkpoint genes demonstrate that the major PD-L1 signal was provided by tumor cells of the basal/squamous subtype, suggesting alterations in tumor cells and TME by tipifarnib could be reversed by PD-1/PD-L1 ICIs.

A serial personalized strategy based on the HRASQ61R addictive mutation and immunosuppressive TME, which are associated with tumor relapse and progression, resulted in favorable clinical responses to tipifarnib and atezolizumab. However, atezolizumab monotherapy did not induce complete remission in the patient, suggesting the requirement of combinatorial immunotherapeutic strategy. Targeted drugs can induce rapid tumor death and lysis with the expression of antigens and neoantingens, which, in turn, could enhance the efficacy of ICIs [92], and the immune TME can act as a source of resistance to MAPK pathway–targeted therapy [89, 90]. Importantly, multiple inhibitory ligand–receptor interactions on T cells (PD-L1 and PD-1, poliovirus receptor and TIGIT, MHC class II and LAG3, and CD80/CD86 and CTLA4) were predicted by analysis of signaling networks of BC159-T#3. The additional inhibitory immune checkpoints that are often expressed in TME include LAG3, TIGIT, and V-domain Ig suppressor of T cell activation, and ongoing clinical trials are starting to investigate the safety and efficacy of combinations of ICIs in advanced solid tumors, including advanced MIUBC [60, 66–68].

The limitations of the present study are the use of PDX platform for a single case, requiring future validation of our strategy using a wide range of refractory tumors in more optimized translational models. In addition, modeling the immune response and rational immune approaches in vivo that rely on data from immunodeficient mice models bear great challenges for the interpretation of our results. The obstacles of translating discoveries from murine experimental data to clinical applications originate from the differences in the organization of the immune system of both species attributed to variations in the protein expression and signaling in the immune systems between mice and humans [93, 94]. Further research on clinically relevant resources and platforms including human primary tumor infiltrating stromal/immune cells derived from MIUBCs, humanized mice, and cancer organoid models [95, 96] are essential to consolidate our hypothesis and to develop novel combination therapeutics for co-targeting human tumor cells and human TME. Ongoing and future clinical trials to determine whether combination therapies targeting other inhibitory pathways, as either doublets or triplets in concurrent or sequential treatment strategies, will provide additional clinical benefits, thus supporting the utility and validity of our scRNA-seq approach.

## Conclusions

Due to the complex and expensive process, generation of PDXs and single-cell RNA sequencing could not be applied in the real-world clinic. Even in the rare case we succeed in the application, we may not be able to investigate the clinical response to the potential combination strategy due to rapid deterioration of the patient’s health. These limitations make the current study more valuable, as we confirmed the efficacy of the treatment choices based on the PDX and single-cell RNA sequencing data. More importantly, the findings here may have a general applicability to cancer patients with activating HRAS mutations. Our findings show the potential of scRNA-seq in discovering precise treatment regimens for overcoming treatment failure to conventional monotherapies and also provide novel insights into unmet clinical needs for effective personalized treatments in a wide range of refractory advanced cancers.

## Supplementary information

**Additional file 1: Table S1.** Somatic variant profiles. **Table S2.** Copy number alteration profiles. **Table S3.** Differentially expressed genes between vehicle and tipifarnib treated mouse fibroblasts from BC159T-#3 PDX. **Table S4.** Differentially expressed genes between vehicle and tipifarnib treated mouse macrophages from BC159T-#3 PDX. **Table S5.** Differentially expressed genes between vehicle and tipifarnib treated mouse T cells from BC159T-#3 PDX.

**Additional file 2: Figure S1.** In silico separation of tumor cells from non-tumor cell types in the scRNA-seq data. **Figure S2.** Identification of human cell types using well-known cell markers. **Figure S3.** Tumor weight change by tipifarnib treatment. **Figure S4.** Quality control of total cells and separation of human cells and mouse cells. **Figure S5.** Potential target drug resistance mechanism in tumor cell heterogeneity. **Figure S6.** Identification of mouse cell types using well-known cell markers. **Figure S7.** Identification of mouse fibroblast subtypes using well-known cell markers. **Figure S8.** Identification of mouse macrophage subtypes using well-known cell markers. **Figure S9.** Identification of mouse T cell and NK cell subtypes using well-known cell markers. **Figure S10.** Personalized treatment strategy after target drug resistance.

## Data Availability

Raw sequencing data for this case report are available in the European Genome-phenome Archive (EGA) database (EGAD00001005978) [97]. Processed data including scRNA-seq and whole transcriptome sequencing are available in the NCBI Gene Expression Omnibus database under the accession number GSE145140 [98]. Clustering and gene expression for the scRNA-seq can be explored at the interactive website [http://ureca-singlecell.kr]. The TCGA-BLCA dataset referenced during the study [32] are available from the Firehose website [http://gdac.broadinstitute.org/].

## Abbreviations

TME: Tumor microenvironment
scRNA-seq: Single-cell RNA sequencing
MIUBC: Muscle-invasive urothelial bladder cancers
PDX: Patient-derived tumor xenograft
IRB: Institutional Review Board
IHC: Immunohistochemistry
RT: Room temperature
HRAS: Harvey rat sarcoma viral oncogene homolog
CK: Cytokeratin
p: Phosphorylated
ERK: Extracellular signal-regulated kinase
AKT: Protein kinase B
PD-L1: Programmed death-ligand 1
TUNEL: Terminal deoxynucleotidyl transferase-mediated dUTP nick-end labeling
WES: Whole exome sequencing
CNV: Copy-number variation
WTS: Whole transcriptome sequencing
TPM: Transcripts per million
TCGA: The Cancer Genome Atlas
UMI: Unique molecular identifiers
NRAS: Neuroblastoma RAS viral (v-ras) oncogene homolog
PC: Principal component
GSVA: Gene set variation analysis
ICI: Immune checkpoint inhibitor
EMT: Epithelial-to-mesenchymal transition
TUR-BT: Transurethral resection of the bladder tumor
BCG: Bacillus Calmette–Guerin
FTI: Farnesyltransferase inhibitor
IGFBP7: Insulin-like growth factor-binding protein 7
MDK: Midkine
B2M: Beta-2-microglobulin
PI3K: Phosphoinositide 3-kinase
mTOR: Mammalian target of rapamycin
COL14A1: Collagen alpha-1(XIV) chain
CAFs: Cancer-associated fibroblasts
COL14A1 MatrixCAFs: Collagen alpha-1(XIV) chain-expressing matrix cancer-associated fibroblasts
ACTA2: Actin alpha 2, smooth muscle
MyoCAFs: Actin alpha 2, smooth muscle-expressing cancer-associated myofibroblasts
TAM: Tumor-associated macrophage
CTL: Cytotoxic T lymphocyte
Treg: Regulatory T cell
CTLA4: Cytotoxic T lymphocyte-associated protein 4
LAG3: Lymphocyte activation gene-3
TIGIT: T cell immunoreceptor with Ig and ITIM domains
PD-1: Programmed cell death-1
MHC: Major histocompatibility complex
MEK: Mitogen-activated protein kinase kinase
