# Supplementary material for: Single-cell RNA sequencing reveals the tumor microenvironment and facilitates strategic choices to circumvent treatment failure in a chemorefractory bladder cancer patient
Source: Genome Med. 2020 May 27;12:47. doi: 10.1186/s13073-020-00741-6 (PMC7251908; doi:10.1186/s13073-020-00741-6)
Supplement: Supplementary file 2 — Additional file 2: Figure S1. In silico separation of tumor cells from non-tumor cell types in the scRNA-seq data. Figure S2. Identification of human cell types using well-known cell markers. Figure S3. Tumor weight change by tipifarnib treatment. Figure S4. Quality control of total cells and separation of human cells and mouse cells. Figure S5. Potential target drug resistance mechanism in tumor cell heterogeneity. Figure S6. Identification of mouse cell types using well-known cell markers. Figure S7. Identification of mouse fibroblast subtypes using well-known cell markers. Figure S8. Identification of mouse macrophage subtypes using well-known cell markers. Figure S9. Identification of mouse T cell and NK cell subtypes using well-known cell markers. Figure S10. Personalized treatment strategy after target drug resistance. [file 13073_2020_741_MOESM2_ESM.docx]

**Additional file 2: Supplementary Figures**

**Single-cell RNA sequencing reveals the tumor microenvironment and facilitates strategic choices to circumvent treatment failure in a chemorefractory bladder cancer patient**

Hye Won Lee, Woosung Chung, Hae-Ock Lee, *et al*

**
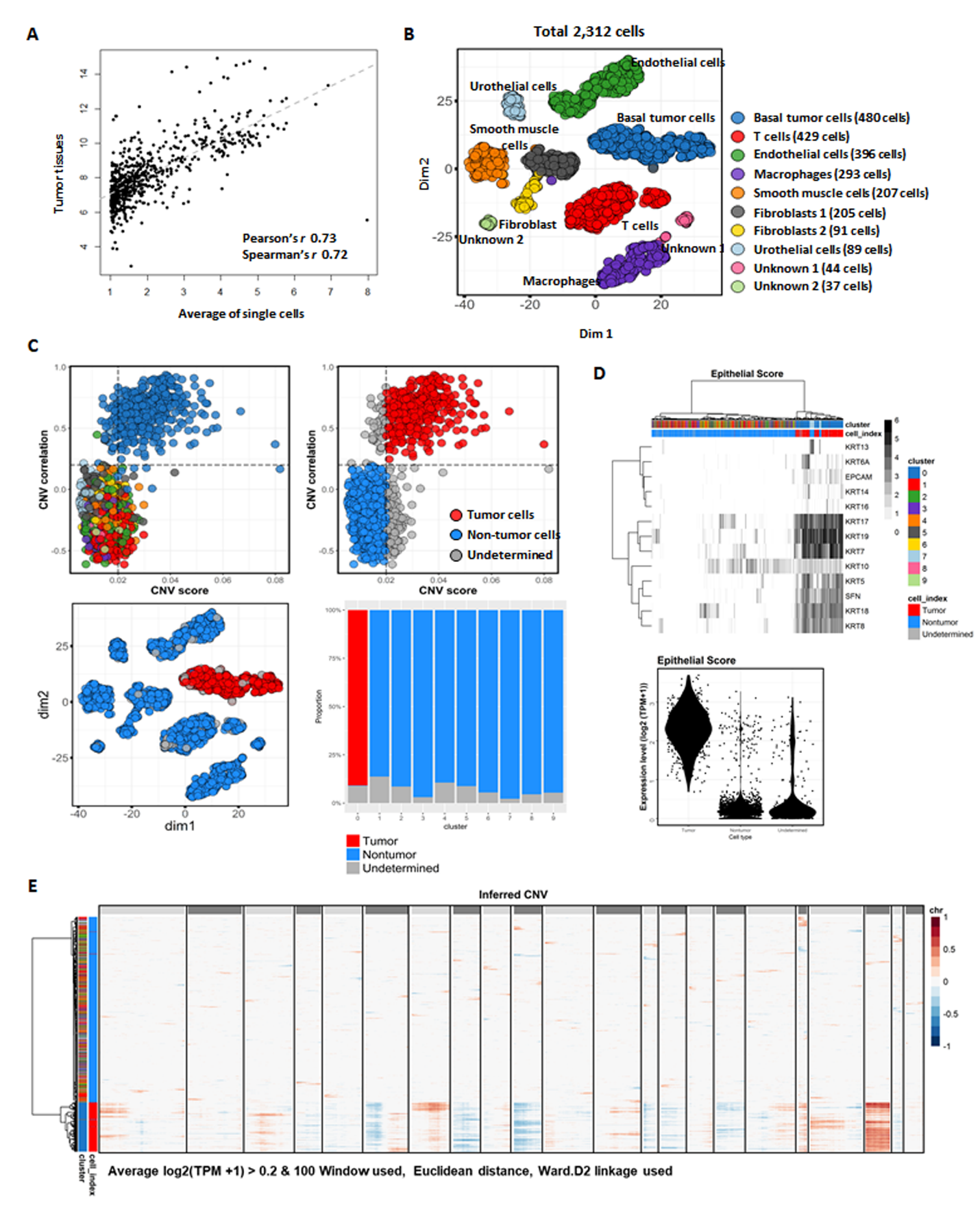
**

**Figure S1** In silico separation of tumor cells from non-tumor cell types in the scRNA-seq data. **a** Average of single-cell expression represents significant correlation with bulk RNA sequencing (Pearson’s correlation coefficient, 0.73, *P* < 2.2e-16; Spearman’s correlation coefficient, 0.72, *P <* 2.2e-16). **b** tSNE plot of 2,312 cells in BC159-T#3 sample before CNV filtration process, color-coded by their graphic-based clusters. **c** Scatter plot showing CNV score versus CNV correlation, color-coded by cell type (top left panel) and tumor classification (top right panel). tSNE plot showing CNV filtration (bottom left panel). Relative cell proportion of tumor cells and non-tumor cells for each cluster (bottom right panel). **d** Heatmap of single-cells with supervised clustering showing the mRNA expression levels of epithelial related genes (top panel). Violin plot of epithelial score for assigned cell types (bottom panel). **e** Hierarchical clustering of inferred CNV scores separating the tumor cell cluster and non-tumor cell type cluster. Euclidean distance with Ward.D2 linkage was used.


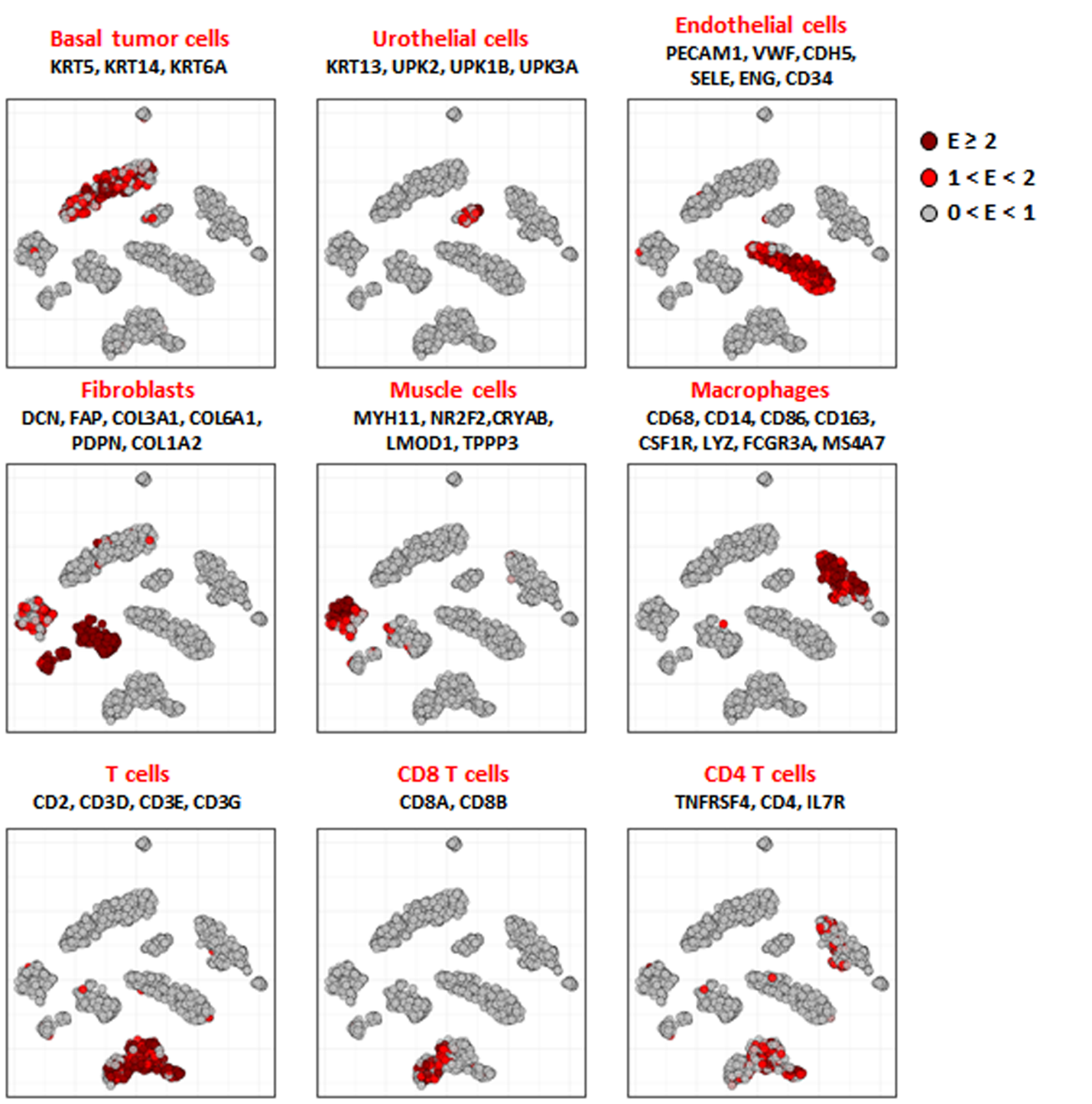


**Figure S2** Identification of human cell types using well-known cell markers. tSNE plots represent the average expression of well-known markers for specific human cell types. Dark red dots indicate cells with high expression (E ≥ 2), light red dots indicate cells with intermediate expression (1 < E < 2), grey dots indicate cells that do not meet the cutoff value.

**
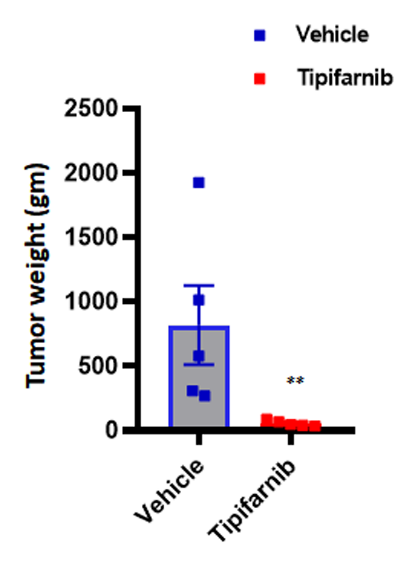
**

**Figure S3** Tumor weight change by tipifarnib treatment. Tumor weight was estimated in BC159-T#3 PDX administered tipifarnib (50 mg/kg) or vehicle as control. A significant effect on tumor weight was observed between tipifarnib and vehicle groups (Student’s *t* test, ***P <* 0.01, Error bar; SD).

**
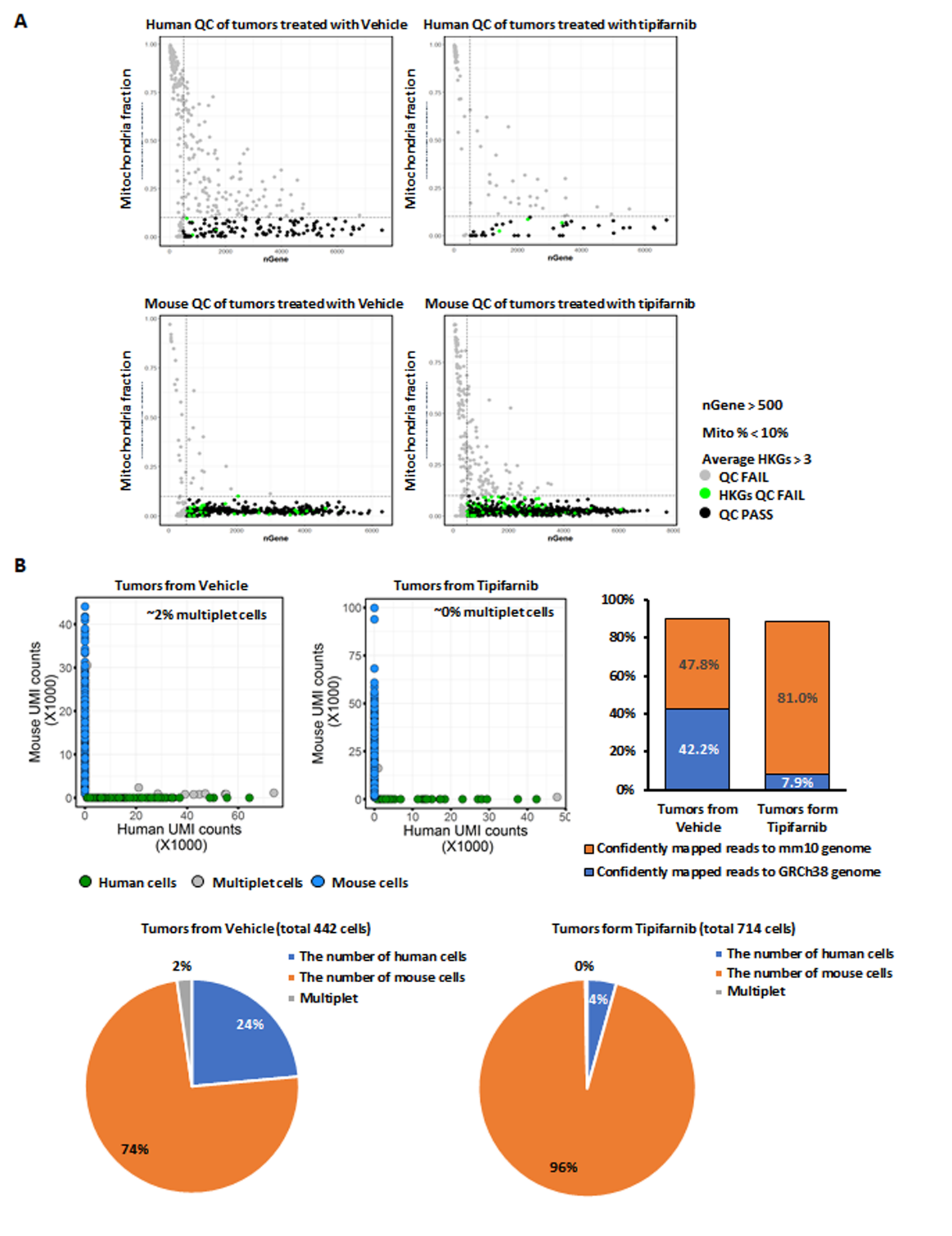
**

**Figure S4** Quality control of total cells and separation of human cells and mouse cells. **a** Scatter plots of the number of genes and mitochondria genome fraction. Horizontal and vertical dash lines indicate the cut-offs at more than 500 detected genes and less than 10% of mitochondria genome fraction. Black dots indicate cells that satisfy all criteria, grey dots indicate cells that do not meet the criteria. The average mRNA expression levels of known housekeeping genes in all cells were calculated, and below 3 were considered as low-quality cells (green dots) and excluded from further analysis. **b** Scatter plots of UMI counts mapped to human and mouse reference genome from PDX that were treated tipifarnib or vehicle. Green dots indicate cells that were human specific, blue dots indicate cells that were mouse specific, 1~2% cells are assigned as multiplet cells (top left panel). Proportion of mapped reads to human genome (GRCh38) or mouse genome (mm10) of PDX that were treated tipifarnib or vehicle (top right panel). Pie charts of the number of human, mouse, and multiplet cells (bottom panel).

**
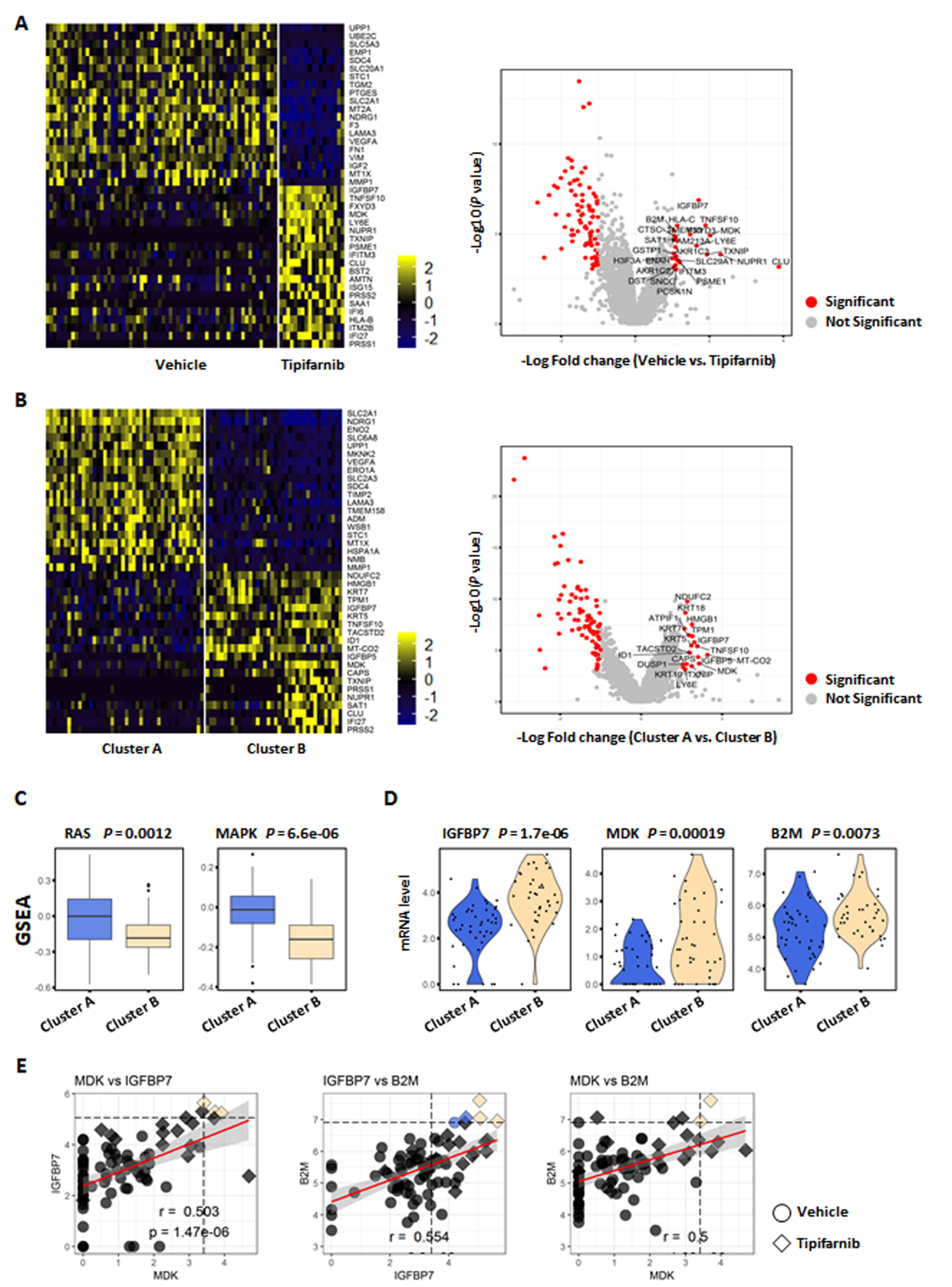
**

**Figure S5** Potential target drug resistance mechanism in tumor cell heterogeneity. **a** Differentially expressed gene expression heatmap of tipifarnib-resistant cells versus control (left panel). Volcano plot of differentially expressed genes between before and after tipifarnib treatment tumor cells. Genes that cause targeted therapy resistance (*IGFBP7*, *MDK*, and *B2M*) are up-regulated in tumor cells after tipifarnib treatment. *P*-values estimated from Student’s *t* test. Genes colored by red are fold-change > 0.8 with *P* < 0.001. **b** Differentially expressed gene expression heatmap of unsupervised clusters of tumor cells from PDX model of BC159T-#3 (left panel). Volcano plot of differentially expressed genes between Cluster A and Cluster B. Genes that cause targeted therapy resistance (*IGFBP7*, *MDK*, and *B2M*) are up-regulated in tumor cells from Cluster B. *P*-values estimated from Student’s *t* test. Genes colored by red are fold-change > 0.8 with *P* < 0.001. **c** The difference of RAS and MAPK pathway activation between Cluster A and Cluster B. Student’s *t* test, *P* = 0.0012, *P* = 6.6e-06 respectively. Each box shows the median and IQR (interquartile range, 25th to 75th percentiles), whiskers indicate the highest and lowest value within 1.5 times the IQR and outliers are marked as dots. **d** Violin plots of significantly up-regulated genes (*IGFBP7*, *MDK*, and *B2M*) in Cluster B for total PDX tumor cells (dots). Student’s *t* test, *P* = 1.7e-05, *P* = 0.00019, *P* = 0.0073 respectively. **e** Pearson’s correlation coefficient between genes that cause targeted therapy resistance (*IGFBP7*, *MDK*, and *B2M*). Horizontal and vertical dashed lines indicate signature score cut-offs at top 5%. The linear regression result is drawn as a solid line. The colored dots mark cells with high expression signatures for both axis. Cells from Cluster A or B were marked as blue or yellow, cells from tipifarnib-resistant cells or control cells were marked as diamond or circle.

**
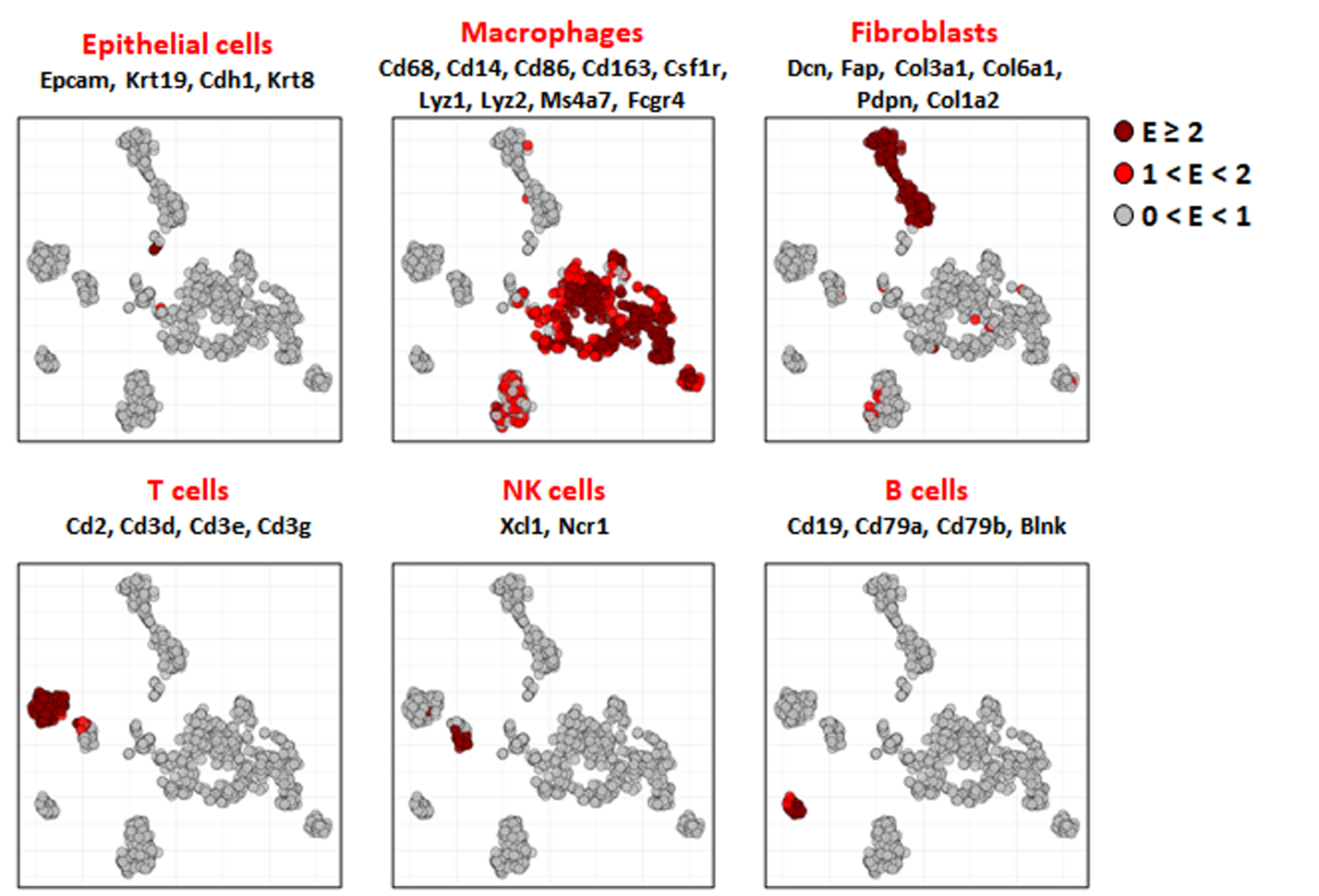
**

**Figure S6** Identification of mouse cell types using well-known cell markers. tSNE plots represent the average expression of well-known markers for specific mouse cell types. Dark red dots indicate cells with high expression (E ≥ 2), light red dots indicate cells with intermediate expression (1 < E < 2), grey dots indicate cells that do not meet the cutoff value.

**
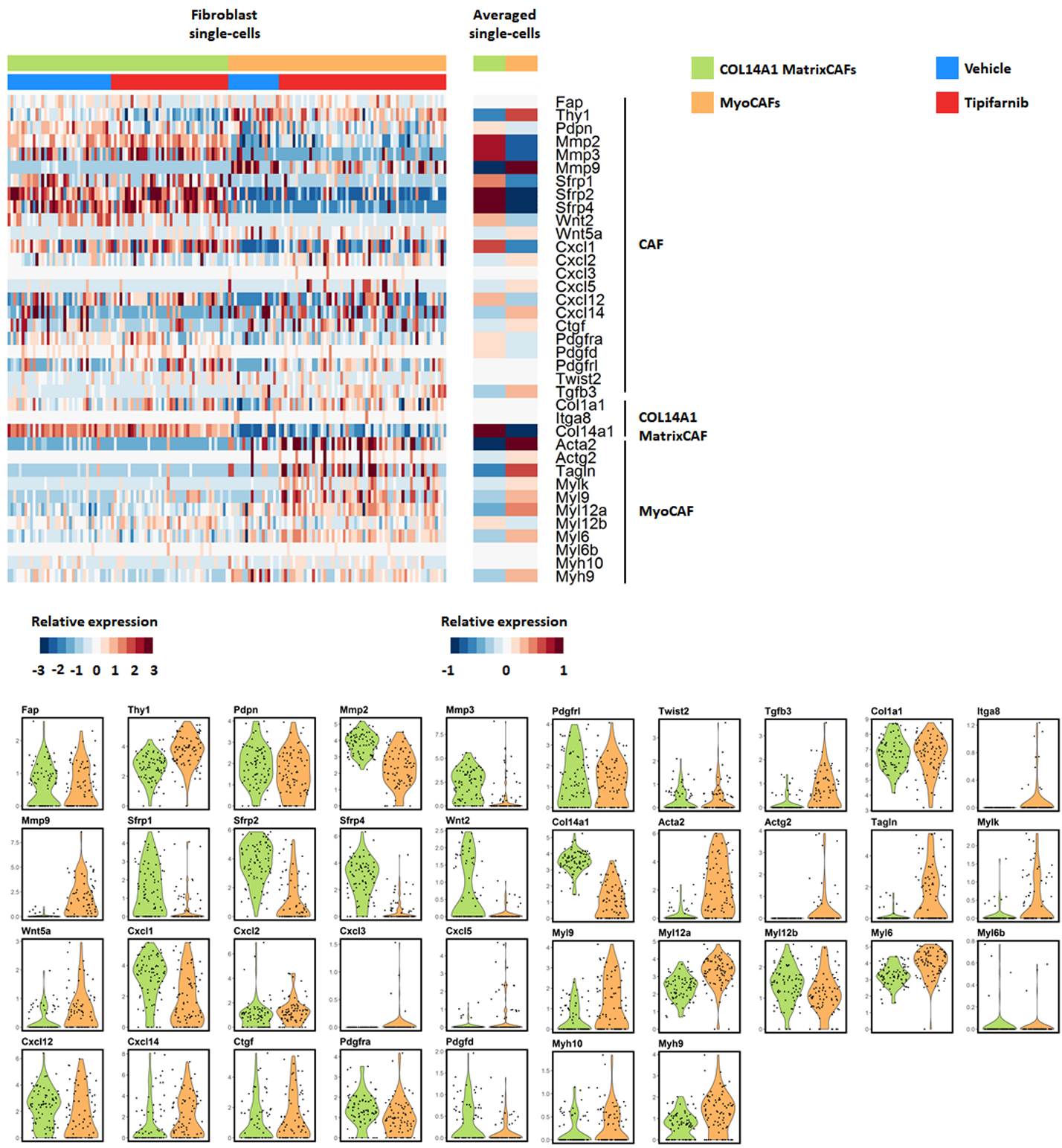
**

**Figure S7** Identification of mouse fibroblast subtypes using well-known cell markers. Heatmaps (top panel) and violin plots (bottom panel) of single-cells represent the mRNA expression levels of well-known marker genes of fibroblast subtypes.

**
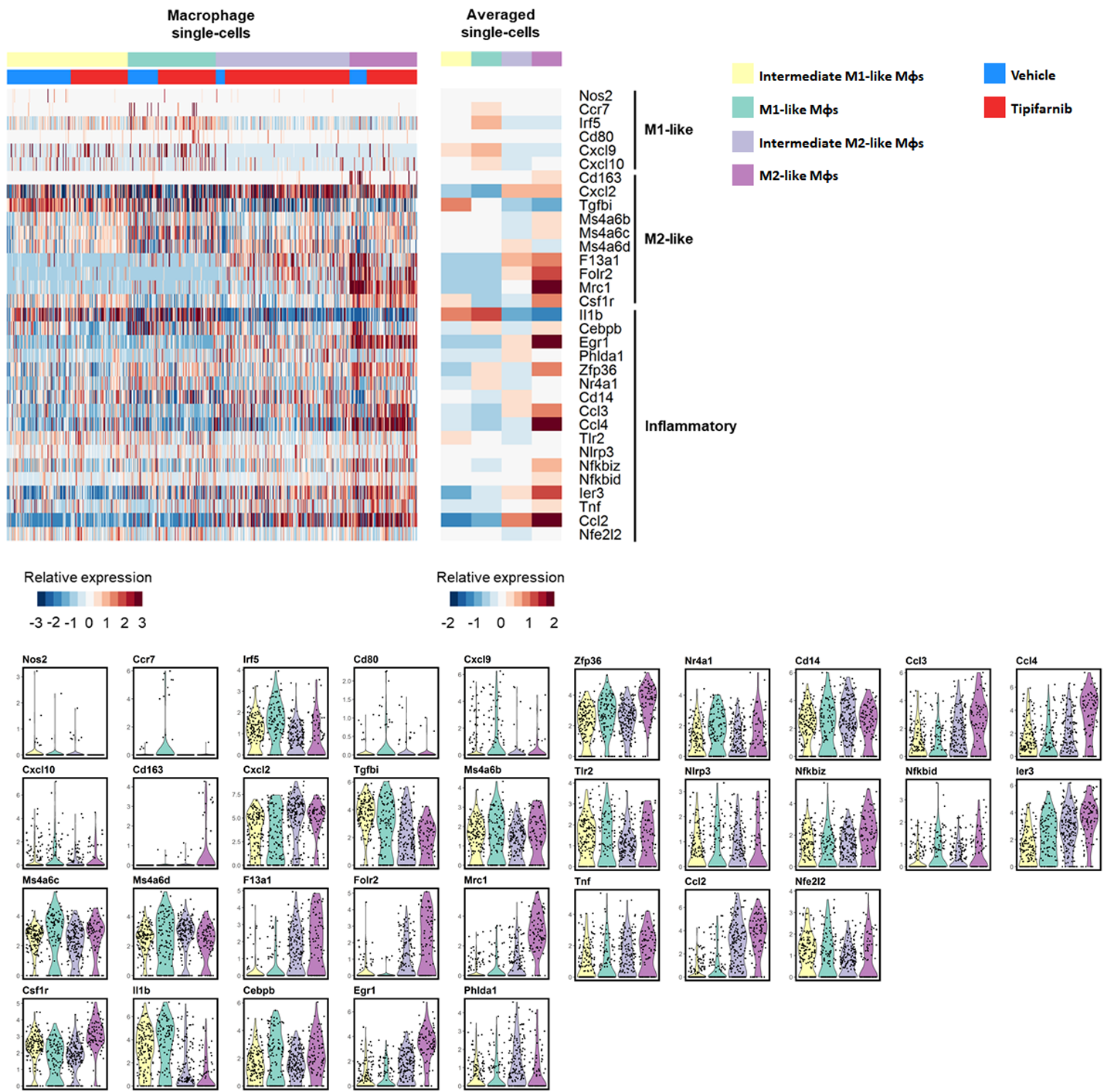
**

**Figure S8** Identification of mouse macrophage subtypes using well-known cell markers. Heatmaps (top panel) and violin plots (bottom panel) of single-cells represent the mRNA expression levels of well-known marker genes of macrophage subtypes.

**
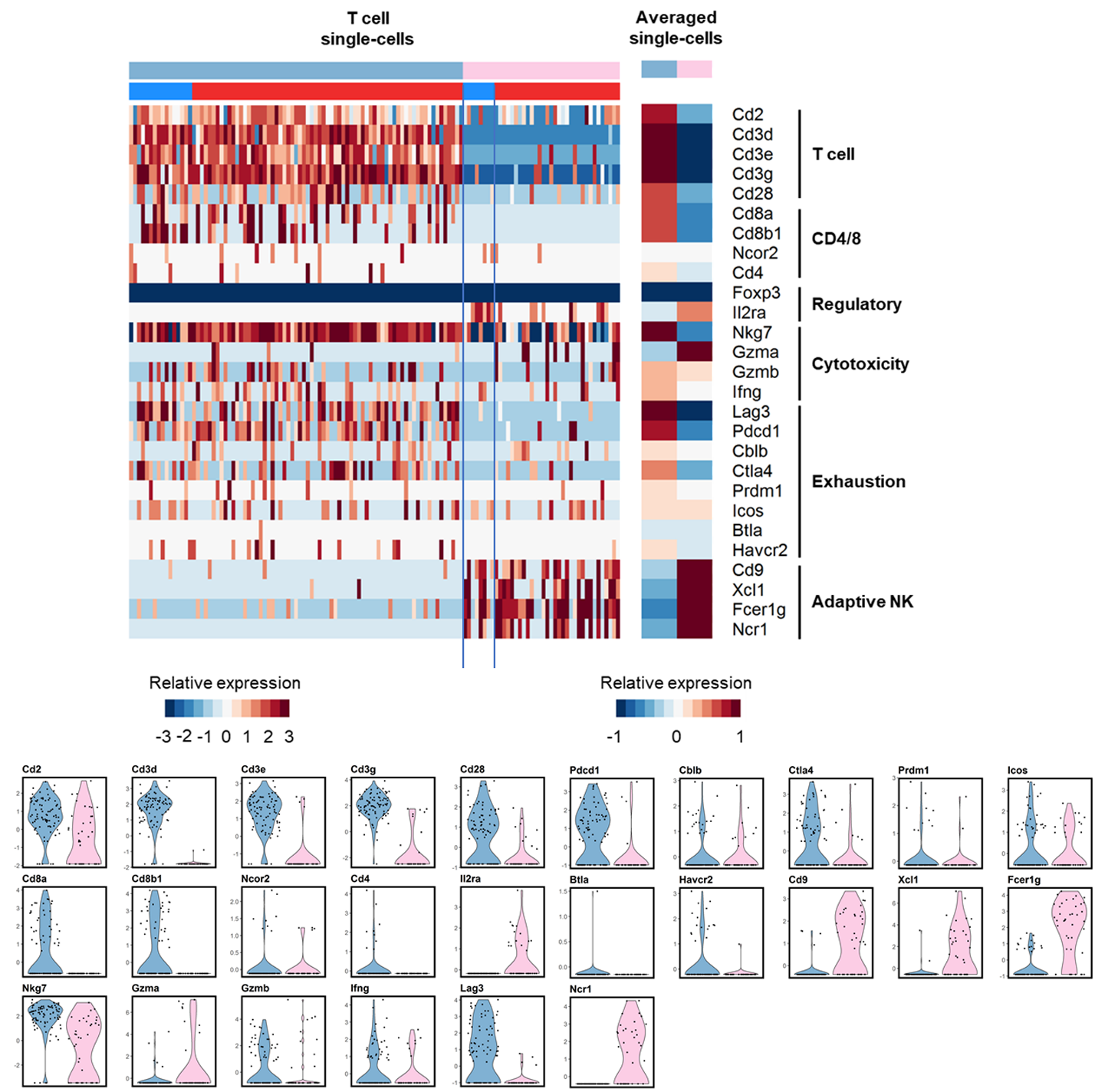
**

**Figure S9** Identification of mouse T cell and NK cell subtypes using well-known cell markers. Heatmaps (top panel) and violin plots (bottom panel) of single-cells represent the mRNA expression levels of well-known marker genes of T cell and NK cell subtypes.

**
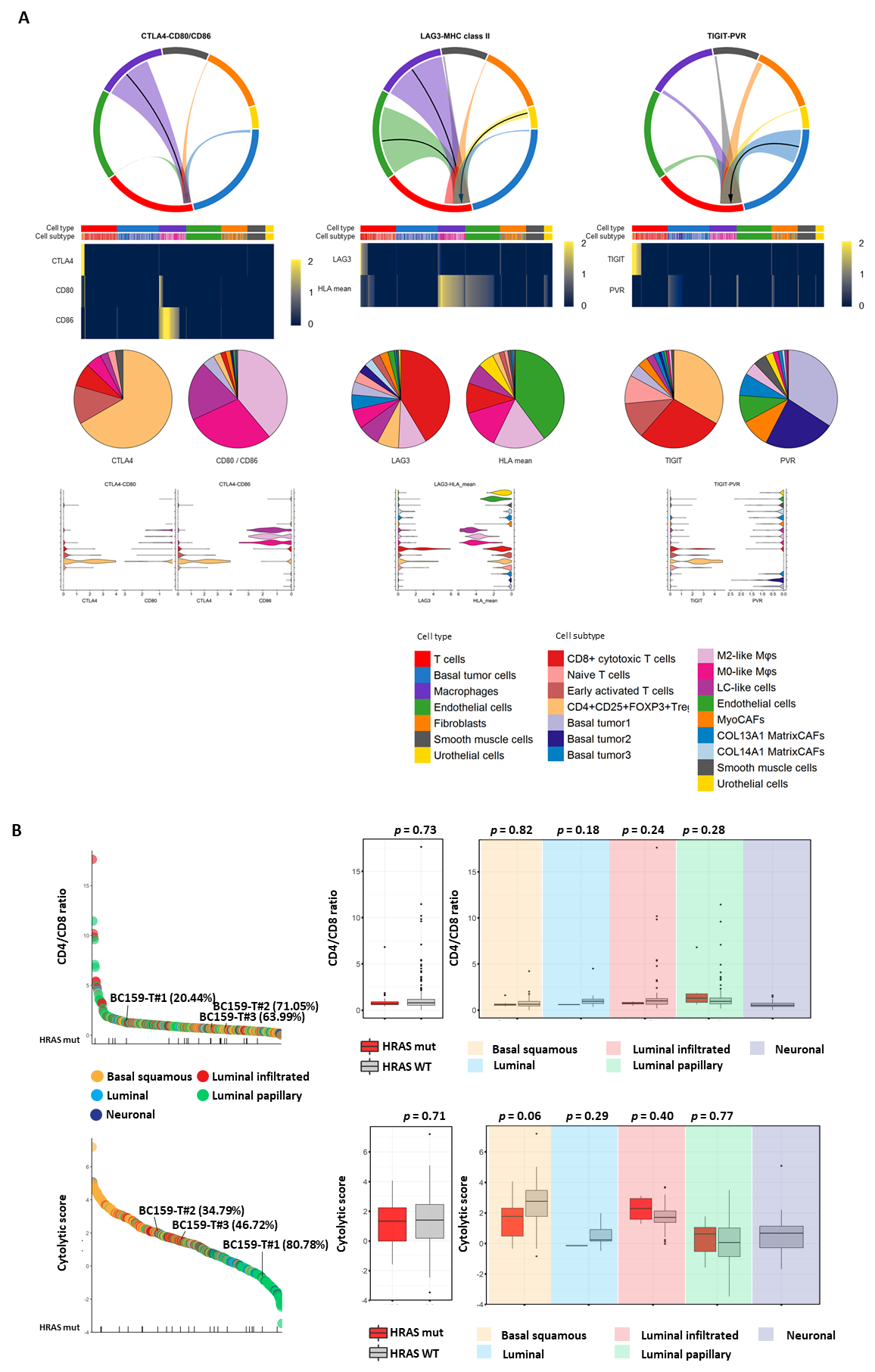
**

**Figure S10** Personalized treatment strategy after target drug resistance. **a** Receptor-ligand interaction between pairs of immunotherapy target (CTLA4-CD80/CD86, LAG3-MHC class II, and TIGIT-PVR) of total human cells (BC159-T#3). Circle plot showing fraction of cells that express ligand genes (CD80/CD86, MHC class II related genes, and PVR) connected to fraction of T cells that express receptor genes (CTLA4, LAG3, and TIGIT). Arrows indicate the direction of interaction (from ligand to receptor) that expresses more than 10% of the ligand genes (top panel). Heatmap of single-cells showing the mRNA expression levels of receptor-ligand pairing genes (first middle panel). Pie charts demonstrating the cell composition that express receptor-ligand pairing genes (second middle panel). 2D-violin plots represent each interaction of receptor-ligand pairing genes (bottom panel). **b** CD4/CD8 ratio and Cytolytic scores are plotted for BC159-T#1, #2, #3, and TCGA-BLCA samples. BC159T-#3 sample ranked in top 63.99 and 46.72 percent, respectively. All samples are colored by molecular subtype. The samples with HRASQ61R mutation are indicated in the bottom row of vertical ticks (left panel). The difference of CD4/CD8 ratio and Cytolytic scores according to HRASQ61R mutation (right panel). Each box shows the median and IQR (interquartile range, 25th to 75th percentiles), whiskers indicate the highest and lowest value within 1.5 times the IQR and outliers are marked as dots. *P*-values estimated from Student’s *t* test.
